# Urban heat vulnerability: A dynamic assessment using multi-source data in coastal metropolis of Southeast China

**DOI:** 10.3389/fpubh.2022.989963

**Published:** 2022-10-20

**Authors:** Chaowei Wu, Wei Shui, Zhigang Huang, Chunhui Wang, Yuehui Wu, Yinpan Wu, Chengzhi Xue, Yunhui Huang, Yiyi Zhang, Dongyang Zheng

**Affiliations:** ^1^College of Environment and Safety Engineering, Fuzhou University, Fuzhou, China; ^2^School of Public Health, Fudan University, Shanghai, China; ^3^Fujian Meteorological Bureau, Fuzhou, China; ^4^Fujian Meteorological Service Center, Fujian Meteorological Bureau, Fuzhou, China; ^5^Taining Meteorological Bureau, Taining, China; ^6^Department of Geography, McGill University, Montreal, QC, Canada; ^7^Fujian Zhitianqi Information Technology Co., Ltd, Fuzhou, China

**Keywords:** climate change, extreme heat, human-environment system, vulnerability, dynamic assessment, Xiamen City

## Abstract

Extreme heat caused by global climate change has become a serious threat to the sustainable development of urban areas. Scientific assessment of the impacts of extreme heat on urban areas and in-depth knowledge of the cross-scale mechanisms of heat vulnerability forming in urban systems are expected to support policymakers and stakeholders in developing effective policies to mitigate the economic, social, and health risks. Based on the perspective of the human-environment system, this study constructed a conceptual framework and index system of “exposure-susceptibility-adaptive capacity” for urban heat vulnerability (UHV) and proposed its assessment methods. Taking Xiamen City, a coastal metropolis, as an example, spatial analysis and Geodetector were used to explore the spatial and temporal changes, spatial characteristics, and patterns of UHV under multiple external disturbances from natural to anthropological factors, and to reveal the main factors influencing UHV forming and spatial differentiation. Results showed that the exposure, susceptibility, adaptive capacity, and UHV in Xiamen City had a spatial structure of “coastal-offshore-inland”. On the hot day, both the exposure and UHV showed a temporal pattern of “rising and then falling, peaking at 14:00” and a spatial pattern of “monsoonal-like” movement between coast and inland. Coastal zoning with favorable socioeconomic conditions had less magnitude of changes in UHV, where the stability of the urban system was more likely to be maintained. During the hot months, the high UHV areas were mainly distributed in the inland, while coastal areas showed low UHV levels. Further, coastal UHV was mainly dominated by “heat exposure”, offshore by “comprehensive factors”, and inland in the northern mountainous areas by “lack of adaptive capacity”. Multi-scale urban adaptive capacity was confirmed to alter spatial distribution of exposure and reshape the spatial pattern of UHV. This study promotes the application of multi-scale vulnerability framework to disaster impact assessment, enriches the scientific knowledge of the urban system vulnerability, and provides scientific references for local targeted cooling policy development and extreme heat resilience building programs.

## Introduction

Since the industrial revolution, excessive greenhouse gas emissions from human activities have caused frequent extreme climate and weather phenomena, especially extreme heat (1, 2). The Sixth Assessment Report of the Intergovernmental Panel on Climate Change (IPCC) further confirms that even relatively small increments of global warming (+0.5 °C) are highly likely to cause statistically significant extreme temperature changes at global and regional scales (1). Currently, extreme heat not only brings serious economic losses to countries and regions but also is a major challenge to human health. Extreme heat increases the impact of urban heat island effect (3, 4), destroys land transportation infrastructure (5, 6), and affects aviation (7) and energy transportation (8, 9). Moreover, heat waves affect people playing outdoor sports (10), hosting sporting events (11), and also threaten the safety and efficiency of outdoor heat operations (12). The incidence of symptoms such as respiratory distress, heat-sensitive illnesses (13), hospitalizations (14) and even mortality (15, 16) increases significantly during extreme heat events. In the context of global warming, the number of hot days and nights, as well as the duration, frequency, and intensity of heat waves, will continue to increase in the future (1, 17, 18).

The south of China suffers from frequent extreme heat events, in which the average annual number of hot days (daily maximum temperature ≥ 35°C) in eastern China cities ranges from 20 to 30 d, and the average duration of heat waves (consecutive hot days ≥ 3) per year ranges from 20 to 30 d (19). Fujian Province is in the subtropical southeast China. The *2020 Fujian Climate Bulletin* reports that the annual average temperature of the province is 20.6°C, which is the highest on record in the last 60 years; the extreme maximum temperature reaches 41.1°C (20). Xiamen City, a coastal metropolis in Fujian Province on the West Side of the Taiwan Strait, is also one of the special economic zones in China (In May 1980, China designated Shenzhen, Zhuhai, Shantou, and Xiamen as the four special economic zones for the Reform and Opening of China, and over the next 40 years, all four cities have achieved remarkable economic achievements). The Xiamen Meteorological Bureau releases the *Xiamen Annual Climate Report* for the previous year in the first half of each year, and the report shows that the annual average and annual extreme maximum temperatures are both on the rise (Supplementary Figure 1), with the extreme maximum temperature of 39.6°C monitored in 2019, the highest in the past decade. Moreover, the number of hot days in Xiamen City also increased significantly. In 2020, Xiamen Tong'an meteorological monitoring station reported 56 hot days, a historical record since 1956 when meteorological observations were available. Under the influence of the high-rise buildings and urban background winds, the urban heat island effect in Xiamen City may be further intensified, which will lead to the continued expansion of the adverse effects of extreme heat events (21). Coastal metropolitan areas should be important for extreme heat hazard impact assessment, but there is still a lack of knowledge about this type of municipalities, and it remains unclear whether the extent and magnitude of their exposure to extreme heat in the past is still consistent with the present, which makes it difficult to prepare adequately in advance to mitigate the damages caused by extreme heat events.

Heat disasters and hot weather impacts in the context of global change have achieved impressive outcomes in terms of hazard bodies, hazard-bearing bodies, hazard-inducing environments, and health risks (22–25). The intrinsic characteristics of heat hazards, or the relationship between extreme heat and physiological health, have been widely discussed in the “hazard-risk” research framework. However, are all human activities in cities susceptible or vulnerable when exposed to extreme heat events? Can adaptive capacity such as physical health, economic status, welfare level, public investment, and regional resilience building achievements mitigate the negative impacts of extreme heat (26)? Because of the absence of effective assessment of economic and social resilience development and urban planning and building, it remains difficult to comprehensively and accurately understand the comprehensive impacts of extreme heat events on human society.

With the further development of urban heat hazards research, scholars in related fields have shifted from the perspective of “hazard-risk” to “vulnerability of the human-environment system” to understand the impact of regional heat hazards (27). Based on this perspective, coupled systems' response and adaptation to multiple natural and human disturbances can be assessed by combining qualitative and quantitative methods. In a special report on *How Are Climate and Other Environmental Changes Affecting the Vulnerabilities of Coupled Human-Environment Systems?*, the National Research Council of the National Academy of Sciences emphasizes the importance of constructing a multi-scale vulnerability framework to explore geographic differences in exposure, sensitivity, and adaptive capacity, and argues that cross-scale vulnerability mechanisms of coupled systems at local level will be a focus of future research (26, 28, 29). Specifically, the special report notes the practical significance of studies that focus on local scales, dynamic variability of the vulnerability, and individual and regional multi-scale differences in adaptive capacity (26), for example, measurement of spatial differences in UHV, or dynamic identification of vulnerability hotspots using natural environmental and socioeconomic data (28, 30–32). The results obtained from such studies can directly provide decision makers with insights into the extent and magnitude of hazard impacts. However, the previous local-level UHV assessment methods are still difficult to promote, the knowledge of the dynamics of heat vulnerability of urban systems remains unclear, and the forming mechanisms of UHV in specific regions have yet to be revealed (see Supplementary material 1 for detailed description of the above issues).

Under the current increasingly severe extreme heat situation, there is still a lack of systematic studies on urban heat hazard exposure and vulnerability from the perspective of the human-environment system. In this paper, we constructed a general UHV conceptual framework and index system, and took Xiamen City, a coastal metropolis, as an example to observe and summarize the geographical changes and patterns of UHV under multiple external disturbances, identify the cross-scale mechanisms influencing UHV forming and spatial differentiation, and explore strategies for urban resilience building.

## Study area

Xiamen City (24°26' N, 118°04' E) is in Fujian Province, on the southeast coast of China, and an important central city and tourist destination in the Economic Zone on the West Side of the Taiwan Strait. As a coastal metropolis, Xiamen's topography is dominated by plains and hills, with elevations ranging from −22 to 1,175 meters, and the hills and mountains are mostly located in the northern part of Xiamen. Xiamen City is in the subtropical maritime monsoon climate zone, with mild and rainy conditions, and the annual average temperature is around 21°C. Due to the differential temperature airflow of the Pacific Ocean, Xiamen is affected by 4 ~ 5 typhoons per year on average, and most of these typhoons are concentrated in July to September (33).

**Figure 1:**
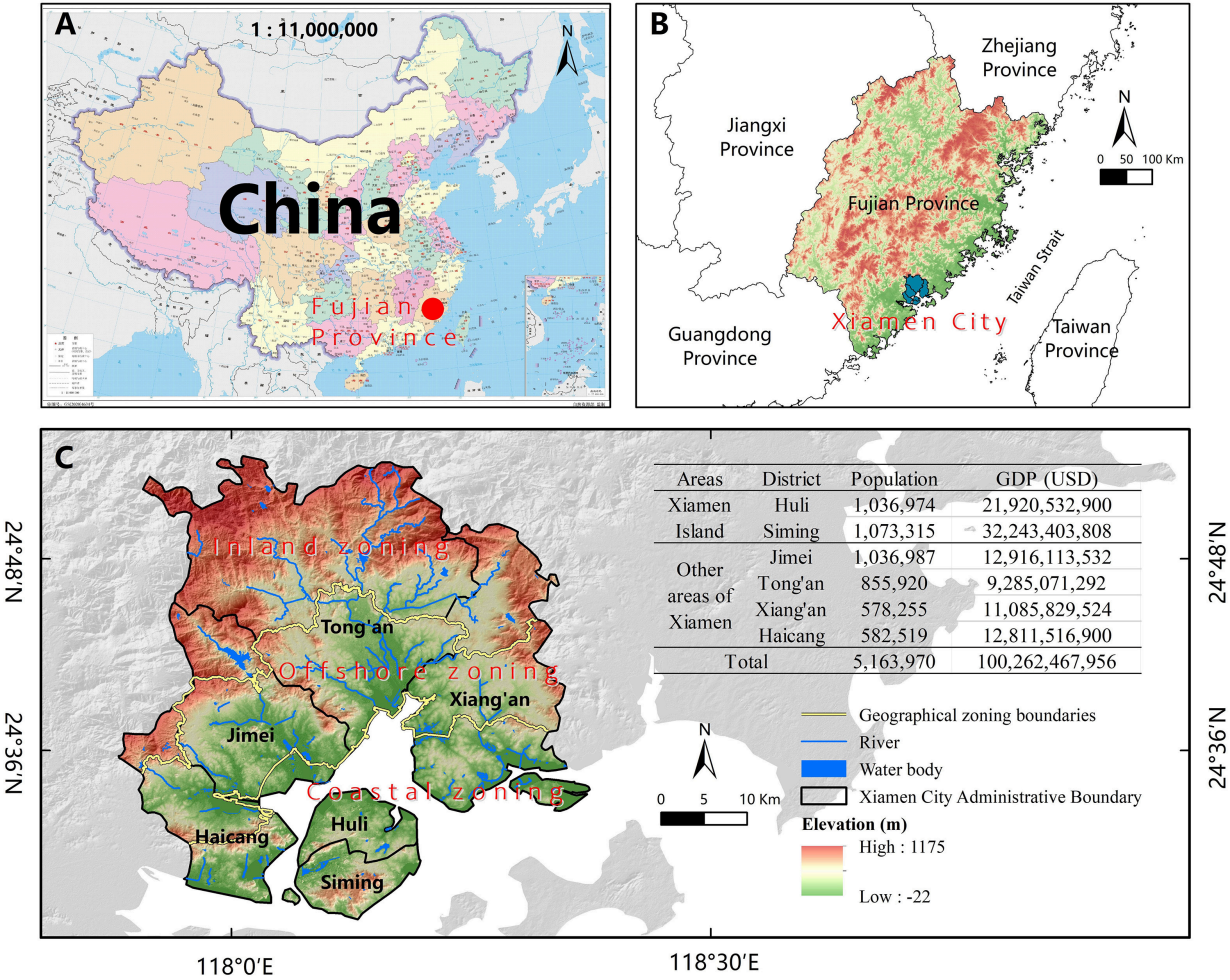
Location map of Xiamen City. **(A)** Location of Fujian Province in China; **(B)** Location of Xiamen City in Fujian Province; **(C)** Xiamen City. Map A source: National Geomatics Center of China (Review No: GS (2020) 4634). GDP source: 2021 yearbook of Xiamen special economic zone. Population source: The seventh National Census.

There are six administrative districts in Xiamen City, among which Siming and Huli are mainly located on Xiamen Island, which are locally called “Dao nei”, while the mainland where Haicang, Jimei, Tong'an, and Xiang'an are located is collectively called “Dao wai” (Figure 1). Xiamen has more than 5 million permanent residents, and many people (> 2.1 million) are distributed on Xiamen Island, which is only 157.98 km^2^ in area and far from the mainland. Based on the administrative boundaries of streets (towns) in Xiamen City, Xiamen Island and the streets (towns) close to the ocean are divided into “coastal zoning”; the streets (towns) far from the coast and dominated by hills and mountains are divided into “inland zoning”; the rest are divided into “offshore zoning”.

## Materials and methods

### Urban heat vulnerability conceptual framework and index system construction

#### Conceptual framework

Vulnerability, a concept that began to appear in the field of natural hazards in the late 1960s, researches focused on the probability of hazards happening, and identification of the extent of their impact (34, 35). In the early 1980s, the *Rights Failure Theory* in the food security field explored the causes of famine vulnerability due to lack of socioeconomic access to the food by focusing on human factors such as welfare levels and social status (35). In the field of geosciences, Timmerman (36) was early interested in vulnerability and argued that vulnerability consists of the magnitude of the impact of a catastrophic event on a system, its resilience to the hazard risk, and its ability to recover from the hazard. Therefore, all the above theories have become important theoretical sources for vulnerability research in different fields. According to Li et al.'s (37) systematic review of the definition of vulnerability in China and abroad, scholars in the field of disaster risk and global change, on one hand, define vulnerability as the probability or magnitude of suffering adverse effects or damages (36, 38), emphasizing the impact or results caused by external disturbances; on the other hand, studies in the field of social sciences in development economics, livelihoods, and poverty consider vulnerability as the ability of individuals or groups to cope, adapt, or recover when exposed to external adverse effects (39), focusing on revealing the driving forces within vulnerability, especially at the human and social aspects (40). Since the new century, researchers have defined vulnerability not only to contain the concepts of exposure and risk in disaster vulnerability, but also to integrate social, institutional, economic, and other human elements into the concept system (38, 41), and have begun to use a multi-scale research framework to explore vulnerability along the path of coupled systems, regarding vulnerability as a multi-element, multi-dimensional, and interdisciplinary concept system (37). In our study, both the comprehensive effects of multiple external disturbances on system vulnerability and the elements, structure, and function of the vulnerable system, as well as the interaction between the vulnerable system and the external environment, are considered.

Urban areas are a human-environment system with poor ecological stability and high vulnerability to natural disasters (e.g., extreme heat, floods, droughts) (38, 42, 43). Because of the general characteristics of human-environment systems, such as complexity, non-linearity, and diversity of spatial and temporal scales, social vulnerability caused by extreme heat faces difficulties in being measured (44). The IPCC's report provides insight into quantifying extreme heat vulnerability in urban areas. The *Climate Change 2022: Impacts, Adaptation and Vulnerability* (45) notes that both the scientific community and society should pay attention to the impact of human activities on global environmental change, as well as the issue of human society's response to global change. Therefore, it is expected to clarify the interaction between the extreme heat exposure and the natural environment and socioeconomic conditions, and to identify the changing characteristics and patterns of heat vulnerability before comprehensive knowledge of the forming mechanism of heat vulnerability in urban areas can be found (26, 30, 46–48). Currently, the IPCC have guided most scholars to deconstruct the heat vulnerability of urban systems in three dimensions: heat exposure, heat sensitivity, and heat adaptive capacity (26, 35, 49–51). Elements including exposure, sensitivity, susceptibility, and adaptive capacity have also become common terms that frequently appear in the definition of heat vulnerability of coupled systems or urban areas (28, 30, 46, 52–54).

**Figure 2:**
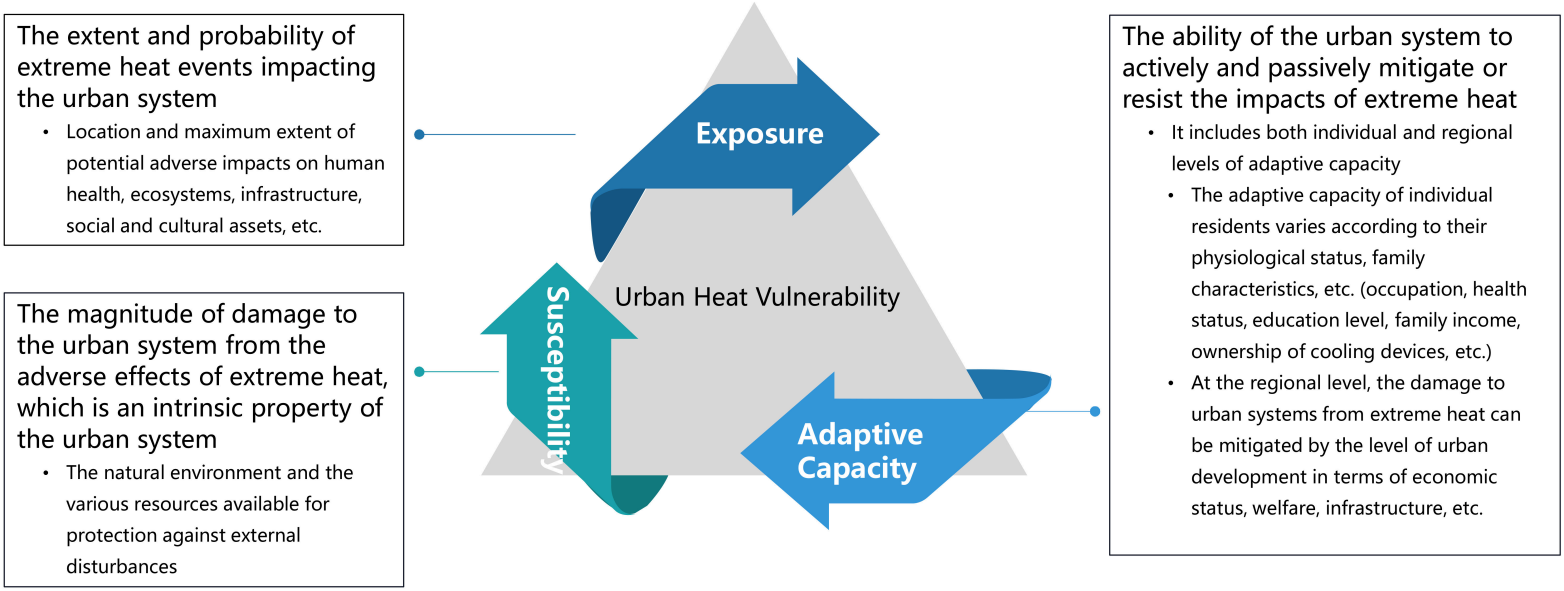
Conceptual framework for UHV.

In summary, the conceptual framework of UHV should represent the impact of urban systems on heat hazards, highlight the multi-scale natural and socioeconomic attributes of the system, and reflect the differences in the ability of individual, households, and regional subjects to cope with and adapt to extreme heat hazards. In this study, UHV from a human-environment system perspective was defined as the probability of a coupled system suffering multiple adverse impacts from heat hazards and the magnitude of its damages, as well as the multi-scale adaptive capacity of people or systems to cope with their impacts. Based on related research results (28), this study proposed and constructed a conceptual framework of UHV consisting of three elements: exposure, susceptibility, adaptive capacity. The concepts of three elements are defined as follows, and their basic components are illustrated in Figure 2.

- Exposure: the extent and probability of extreme heat events affecting the urban system (28, 29, 47, 55);
- Susceptibility: the magnitude of damages to the urban system from the adverse effects of extreme heat, which is an intrinsic property of the urban system (28, 48, 56);
- Adaptive capacity: the ability of the urban system to actively and passively mitigate or resist the impacts of extreme heat (28, 46, 47, 57, 58). It includes both individual (28, 30, 58, 59) and regional (19, 28, 46, 58) levels of adaptive capacity.

#### Index system

Based on the conceptual framework of UHV, this study proposed a set of index system (Table 1). The selection of component indicators of exposure, susceptibility, and adaptive capacity is described as follows:

**Table 1:** Index system for UHV.

**Objectives**	**Elements**		**Evaluation indicators**	**Abbreviation**
Urban Heat	Exposure	Source of exposure	Temperature	TP
Vulnerability			Humidity	HD
		Amount of exposure	Population distribution	POP
	Susceptibility		Land cover	LC
			Elevation	DEM
			Distance from the coastline (adjustable)	CL
			Distance from the water body	WB
	Adaptive Capacity	Individual level	Proportion of resident population aged ≧ 65	POP_65
			Proportion of resident population aged ≦ 14	POP_14
			Proportion of resident population with high school or higher education level	HS
			Per capita disposable income of residents	IC
		Regional level	Supply level of cooling facilities	CF
			Supply level of medical & health facilities	HF

##### Exposure

In this study, heat exposure was considered measured by the amount of exposure of the heat hazard-bearing body (representing the probability of heat exposure) and the spatial and temporal characteristics of the heat hazard body (representing the extent of heat exposure). The urban system was a heat hazard-bearing body, and it has been found that the probability of heat exposure of urban systems is higher in densely populated areas (28, 53), thus we used the population distribution to reflect the amount of heat exposure of urban systems. Some studies have used monitoring data such as the duration of hot days at meteorological stations (19, 60) or near-surface air temperature based on thermal infrared remote sensing to reflect the spatial and temporal characteristics of the heat hazard body (28, 61). Currently, the assessment of climate change based on the synergistic effects of environmental temperature and humidity has become a hot topic (62, 63), and high temperature accompanied by high humidity in the surrounding air reduces the human body's ability to sweat and dissipate heat, which may cause heat stroke or even death (64). Therefore, temperature and humidity were used together to reflect the spatial and temporal characteristics of heat hazard body, which in this study also represented the heat exposure stress faced by urban residents.

##### Susceptibility

In order to take targeted management measures to minimize losses from heat disasters, specific characteristics of the inherent susceptibility attributes of urban systems should be distinguished, which helps to identify urban areas that suffer more impacts and losses. Human activities profoundly influence the type, structure, and quantity of land cover (65), and thus different land cover types in this study represent differences in the intrinsic attributes of urban systems under the influence of human activities. Elevation is the main natural factor influencing the population distribution in China, especially in the eastern part of the country, where the population size at high altitude is small (66) and consequently the probability of heat exposure is low. Lakes, rivers, coasts, bays are confirmed to have significant cooling effect (67, 68), so in this study different distances from these areas were used to reflect differences in the heat susceptibility attributes of the urban system. Combining the characteristics of coastal metropolis, land cover, elevation, water bodies, and coastlines were selected as indicators for measuring urban heat susceptibility.

##### Adaptive capacity

At the individual level, it has been confirmed that due to weaker body regulation or poorer physiological conditions, the elderly, children, and people with heat-sensitive illnesses will experience greater health stress than the general population under equivalent conditions (64, 69). Groups with low or no income who are economically disadvantaged and cannot afford cooling equipment may also face increased heat health risks (28, 30, 52, 70). Educational level affects an individual's ability to adapt to the hot weather. In general, more educational experiences are beneficial for individuals to judge hot weather situations and take timely response measures (28, 30, 48, 59). At the regional level, the development level of community and regional infrastructure, the level of spatial allocation of medical assistance, and the supply level of cooling services (e.g., heat information campaigns) reflect the ability of urban systems to mitigate and adapt to heat stress (30, 48, 57, 71). In this study, the adaptive capacity of urban systems at multiple scales was measured by a combination of the following indicators: age, education level and income at the individual level, and spatial availability (location and quantity) of cooling and health facilities at the regional level.

### Methodology for assessing urban heat vulnerability

#### Urban heat vulnerability model

Based on the conceptual framework and index system of UHV, this study used the multiplication and division method to construct an assessment model of “exposure-susceptibility-adaptability” (Equation 1). The multiplication and division method has been widely acknowledged by most scholars and can effectively reflect the synergistic relationship between the elements of the indicators (28). Because of the different properties and scales of the indicators within each element, all indicators need to be normalized using the linear function normalization method (Equation 2).


(1) $$\begin{array}{l} UHV=E\times V/AC \end{array}$$

(2) $$X^{*}=\;\{\begin{array}{cc} \begin{array}{l} \frac{X-X_{min}}{X_{max}-Xmin},\\ \frac{X_{max}-X}{X_{max}-X_{min}}, \end{array} & \begin{array}{l} Positiveindicators\\ Negativeindicators \end{array} \end{array}$$

where *UHV*, *E*, *V*, *AC* represent UHV, exposure, susceptibility, and adaptive capacity, respectively; X^*^ represents the normalized value of each indicator; *X*, *X*_min_, *X*_max_ represent the original, minimum, and maximum values of each indicator, respectively.

*E*, *V*, *AC* are combined using the weighted summation method (Equation 3).


(3) $$\begin{array}{l} Z_{i}=\overset{m}{\underset{j=1}{\sum }}a_{ij}\times X_{ij}^{*} \end{array}$$

where *Z*_*i*_ is the combined weighted value of *i* element; *a*_*ij*_ is the weighting factor of *j* indicator of *i* element; $X_{ij}^{*}$ is the normalized value of *j* indicator of *i* element.

For the future application of the assessment methodology in other regions, the weighting coefficients of the indicators were determined using an expert group decision method combining hierarchical analysis and the Delphi method (28). When the application of the assessment methodology is extended to cities with different natural environment and socioeconomic characteristics, it is necessary to organize experts to re-weight the above indicators.

Supplementary material 2 contains the characterization and pre-processing methods of the component indicators of the elements, and Supplementary material 3 contains the calculation results of the expert group decision for Xiamen City.

#### Levels of urban heat vulnerability

According to *the National Technical Specification for Weather Hazard Risk Assessment (High Temperature)* (FXPC/QX P-06), and considering the requirement for the assessment model to be easily replicated in other cities, the standard deviation method was used in this study to classify the grade thresholds of exposure, susceptibility, adaptive capacity, and UHV (Table 2).

**Table 2:** Levels of UHV.

**Levels**	**Classification description**	**Explanations**
I	Raster value ≤ Mean – σ	Lowest
II	Mean – σ < Raster value ≤ Mean – 0.5σ	Low
III	Mean – 0.5σ < Raster value ≤ Mean + 0.5σ	Medium
IV	Mean + 0.5σ < Raster value ≤ Mean + σ	High
V	Raster value > Mean + σ	Highest

Mean: Average of non-zero-valued raster in the region; σ: Standard deviation of non-zero-valued raster in the region.

### Spatial analysis methods

In this study, ESRI ArcGIS 10.5 (Environmental Systems Research Institute, Redlands, CA, USA) regional statistical tool was used to explore the temporal change of exposure and UHV during the heat wave in Xiamen City and its six administrative districts. The average center tool was used to explore the spatial changes in the characteristics of the average centers of UHV and exposure for each hour in Xiamen City during the hot day.

In order to explore the spatial and temporal patterns of heat exposure and UHV in Xiamen City on the hot day, the layer overlay method was used to calculate the level differences of heat exposure and UHV from 9:00~14:00 to 14:00~20:00, respectively, and the area of the level change. Spatial autocorrelation (Global Moran's I) was used to assess whether the spatial pattern of UHV and exposure increase (or decrease) in Xiamen City on a hot day was clustered, discrete, or random. The global Moran's I statistic was calculated as the Z-score, and the larger the positive value, the more clustered the global UHV and exposure spatial change; the larger the negative value, the more dispersed. Hot spot analysis (Getis-Ord Gi^*^) was used to identify significant clustering areas (hot and cold spots) of UHV and exposure changes, susceptibility, and adaptive capacity in Xiamen City on a hot day, and to explore high and low UHV areas of Xiamen City in the hot month. The higher the positive Z-score, the tighter the clustering for high UHV; the higher the negative Z-score, the tighter the clustering for low UHV. More information about the spatial analysis methods mentioned in this section can be found in the ArcGIS 10.5 help guide (72).

The differences in the spatial distribution of UHV under the interaction of exposure, susceptibility, and adaptive capacity were analyzed by dividing the dominant element type zoning of Xiamen City. The raster-based layer overlay method was used to classify all raster according to the levels of each element. The specific methods are as follows: (1) Exposure dominance: raster with heat exposure level greater than III; (2) Susceptibility dominance: raster with heat susceptibility level greater than exposure level; (3) Lack of adaptive capacity: raster with heat exposure, susceptibility, and adaptive capacity levels less than III; (4) Comprehensive elements dominance: the rest of unclassified raster.

### Geodetector

The Geodetector (73) was mainly used to explore the key influencing factors of UHV forming in the geographic zoning (coastal-offshore-inland) and the spatial statistical zoning (high UHV areas-low UHV areas-non-significant areas) in Xiamen City. The model is based on the spatial heterogeneity hypothesis and is suitable for study areas with strong spatial heterogeneity. It measures the degree of determining power of potential influencing factors on UHV by calculating the q statistic, that is, determining 100 × q% of UHV, which takes values from 0 to 1. The closer the value is to 1, the stronger the determining power on the spatial differentiation of UHV. In this study, the factor detection method of the model was used, and the main calculation method was as follows:


(4) $$q\;=\;1-\frac{1}{n\sigma^{2}}\sum_{i=1}^{n}N_{i}\sigma_{i}^{2}$$

where N is the total number of samples in the regional block, *i* is the number of layers, σ^2^ is the discrete variance of the distribution of UHV in the study area, and $\sigma_{i}^{2}$ is the variance of the distribution of the *i*th layer.

The input factors of the Geodetector were mainly from the index system proposed in the previous section (Table 1), but these input factors were the original unprocessed indicators. According to the instructions for using Geodetector, all potential influencing factors are not required to be standardized, but the dependent variable input to the model must be continuous and the independent variables must be discrete (74). This study used the original indicators as the independent variables of Geodetector were discretized by R 4.0.2 (The R Programming Language), it is a software developed mainly by Robert Gentleman and Ross Ihaka of Oakland University. In this study, we used the package “GD” (75). More information on the discrete results of the influencing factors is available in Supplementary material 4.

### Data source

According to the daily live weather broadcast from the Xiamen Meteorological Bureau (http://fj.cma.gov.cn/xmsqxj/), several heat waves were observed in Xiamen City beginning in late July 2021. In this study, the heat wave events with high intensity in the daily live weather broadcast were selected: August 7–9, 2021.The temperature and humidity data used in this study were obtained from the thin-grid meteorological element numerical forecast products produced by the FZ-MOS system of Fujian Meteorological Bureau. The FZ-MOS system releases forecasts twice a day at 08:00 and 20:00 BST, with the forecast products released at 08:00 each day and the next day forecast products released at 20:00. The spatial resolution of the products is 0.025° × 0.025°. The products used in this study were: (1) hourly level assessment: temperature and humidity products with a temporal resolution of 1 h released at 08:00 BST each day; (2) daily level assessment: products with a temporal resolution of 24 h released at 20:00 BST each day. The average of the results of all daily level assessments in August 2021 was used to explore the geographical detection mechanism of UHV. This study adopted the regulations of the China Meteorological Bureau for hot weather and heat wave (76): a weather phenomenon with a daily highest temperature ≥ 35°C is called a hot weather or a hot day, and hot weather lasting more than three consecutive days is called a heat wave.

Land cover products were obtained from the 1985 to 2020 global 30 m land cover dynamic monitoring products with fine classification system (GLC_FCS30-1985_2020) developed by Liu's team at the Aerospace Information Research Institute, Chinese Academy of Sciences (77). Various POIs were captured by compiling a Python script on the official website of AutoNavi (https://www.amap.com/), in which the search keywords and numbers for cooling facilities were “subway: 72”, “park: 98”, “shopping mall: 38”, “supermarket: 55”, “library: 47”, and the search keywords and numbers for medical facilities were “community health service centers: 100” and “hospital: 37”. The administrative boundaries of streets (towns) in Xiamen City and the 7th census data of Xiamen were obtained from the Xiamen Natural Resources Bureau and the Xiamen Statistics Bureau, respectively. Data sources and descriptions are available in Supplementary Table 1.

## Results

### Spatial and temporal change characteristics of urban heat vulnerability and exposure during a heat wave

In Xiamen City, the spatial distribution of exposure and UHV showed a spatial structure of “coastal-offshore-inland”. Exposure had the spatial distribution characteristic of “coastal high, inland low”, and the exposure level was higher in the coastal areas of Xiamen Island, Jimei, Haicang and the southern part of Tong'an. UHV was characterized by a spatial distribution of “coastal low, inland high”, with higher vulnerability levels in Tong'an and western Xiang'an (figure is available in Supplementary Figure 2).

In the six administrative districts of Xiamen City: (1) the average exposure levels in Huli was the highest, in Xiang'an and Tong'an was low; (2) the average UHV levels in Xiang'an was the highest, in Siming was the lowest; (3) The ranking of the average exposure and UHV levels in the six administrative districts was relatively consistent during a heat wave; (4) the standard deviations of the average exposure and UHV levels for all of Xiamen and its six administrative districts did not show significant differences during a heat wave (tables are available in Supplementary Tables 2, 3).

According to the above results, we first found that the heat exposure and UHV of coastal metropolis were relatively stable at daily level, and there was little difference in their spatial distribution characteristics, which helps to further down-scale analysis of the spatial and temporal patterns of growth (or decreasing) of exposure and UHV at the hours level.

### Spatial and temporal change characteristics of urban heat vulnerability and exposure on a hot day

#### Temporal changes and characteristics

**Figure 3:**
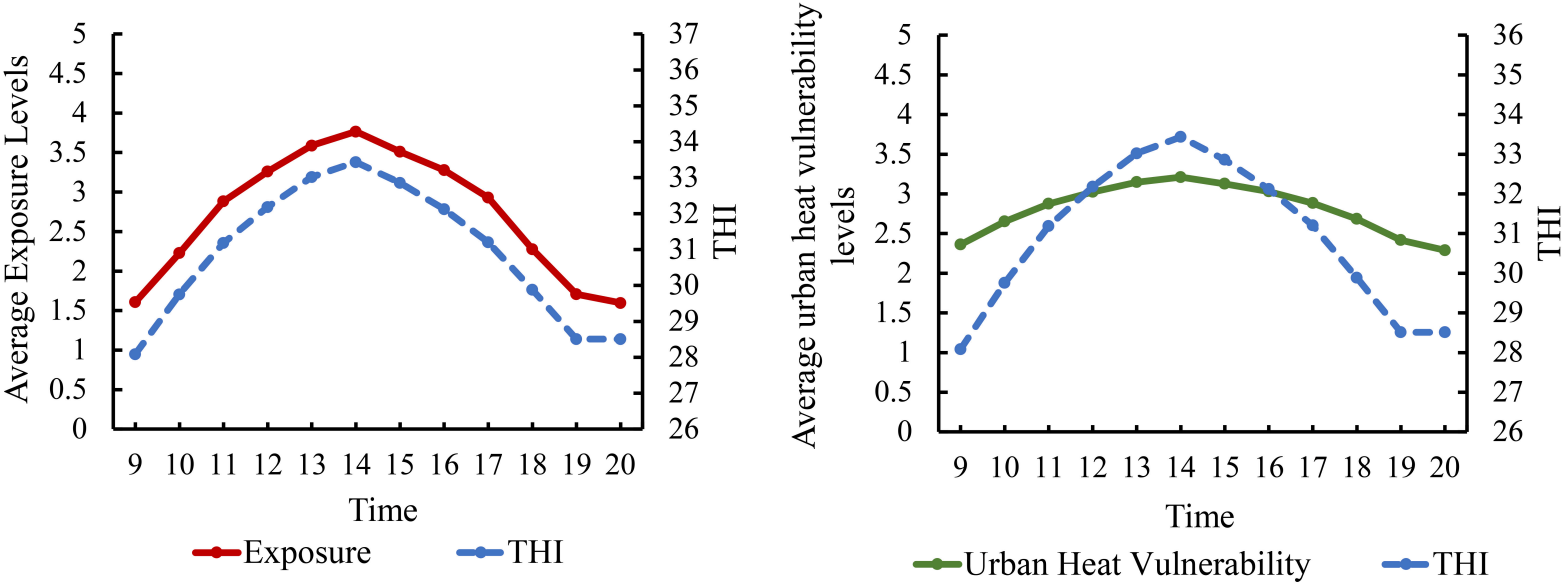
Temporal changes in average exposure and UHV levels on a hot day in Xiamen City. THI: Temperature Humidity Index, details about THI are available in the Supplementary material 2.

The average exposure and UHV levels in Xiamen City from 09:00 to 20:00 on August 7, 2021 showed a temporal change characteristic of “rising first and then falling, peaking at 14:00” (Figure 3), which is consistent with the temporal change trend of Temperature Humidity Index (THI, details about THI are available in the Supplementary material 2). It indicates that to some extent the exposure and UHV were driven by THI during the hot day.

**Figure 4:**
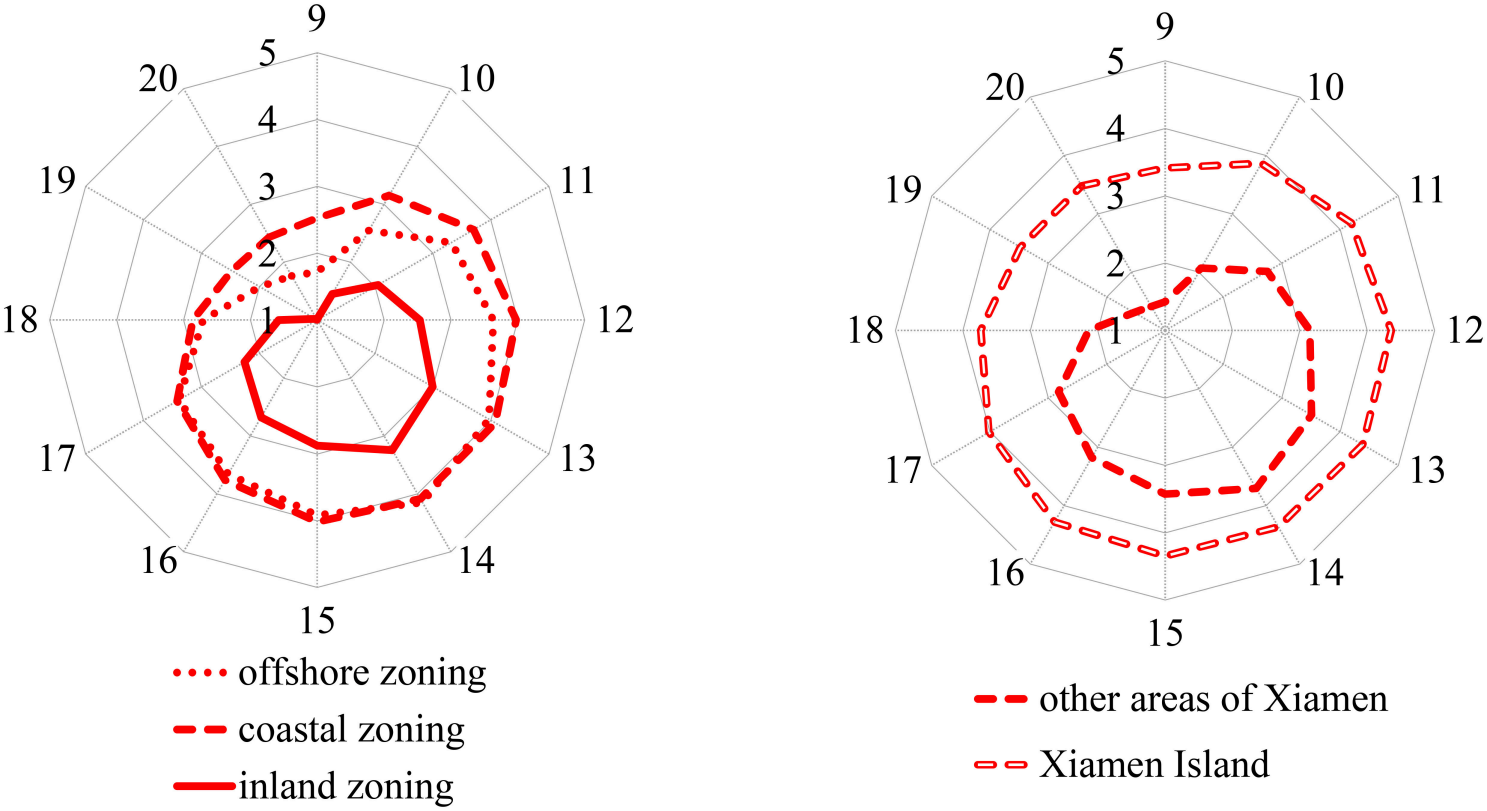
Temporal changes in average exposure levels in coastal, offshore, and inland zonings of Xiamen City on a hot day from 9:00 to 20:00.

**Figure 5:**
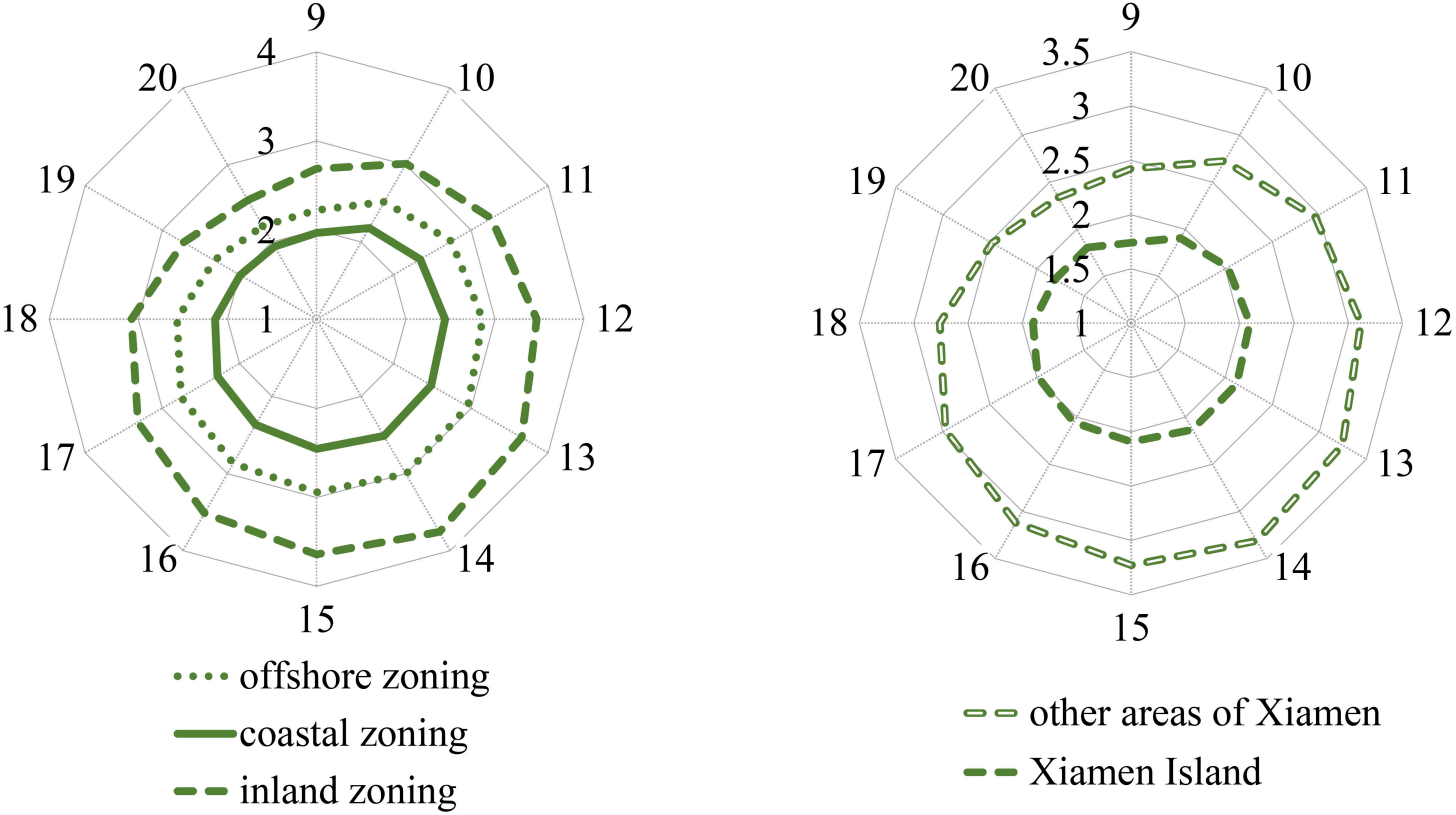
Temporal changes in average UHV levels in coastal, offshore, and inland zonings of Xiamen City on a hot day from 9:00 to 20:00.

Figures 4, 5 show that the average exposure levels in the coastal zoning of Xiamen were higher than those in the inland zoning, but the average UHV levels were opposite; the average exposure levels on Xiamen Island were higher than those outside Xiamen Island, but the UHV was opposite.

#### Spatial changes and characteristics

**Figure 6:**
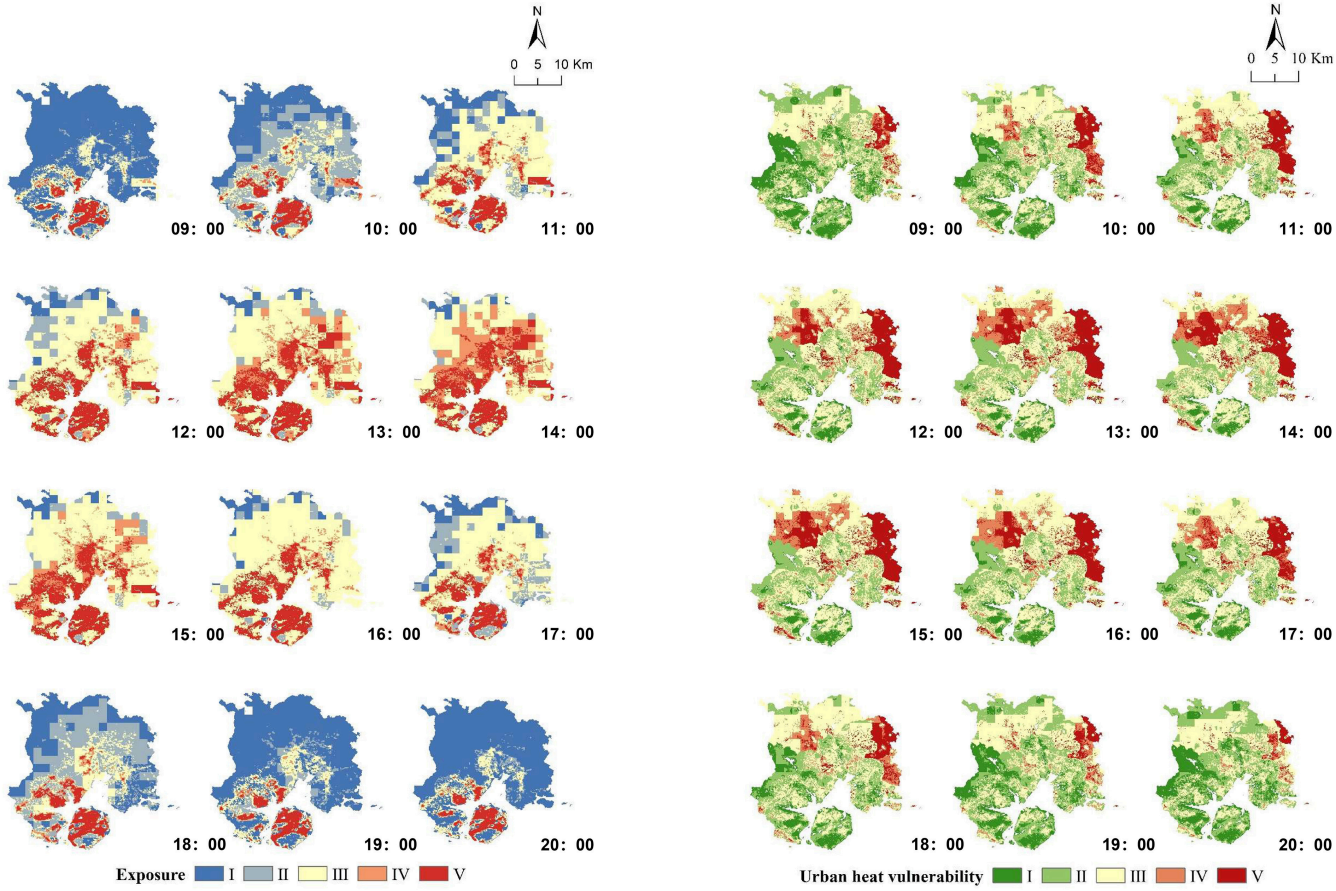
Spatial changes of exposure and UHV on a hot day from 9:00 to 20:00 in Xiamen City.

Figure 6 shows the spatial changes of the exposure and UHV in Xiamen City from 09:00 to 20:00, and it can be observed that exposure has the characteristics of expanding from coastal areas to inland, reaching a peak and then decreasing toward the coast, while UHV has less change on Xiamen Island, but outside the island, it increases from east to west and then decreases toward west.

From 09:00 to 14:00, the average center of exposure moved from southwest to northeast, representing the expanding process of heat exposure from coast to inland; while the average center of UHV moved from northeast to southwest, representing its growing process. After 13:00 and 14:00, the average centers of exposure and UHV move to the southwest and southeast, respectively, representing the decreasing process of both (figure is available in Supplementary Figure 3).

By observing the changes of exposure and UHV on a hot day, we found that they showed a “monsoonal” rather than “random” spatial pattern of change between coastal, offshore, and inland of Xiamen City.

#### Patterns of changes in spatial distribution

**Figure 7:**
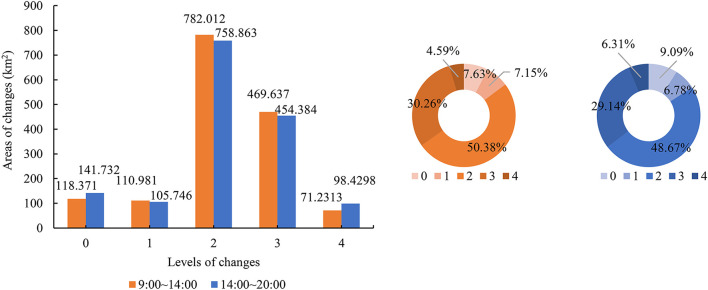
Areas of changes in exposure levels in Xiamen City on a hot day.

**Figure 8:**
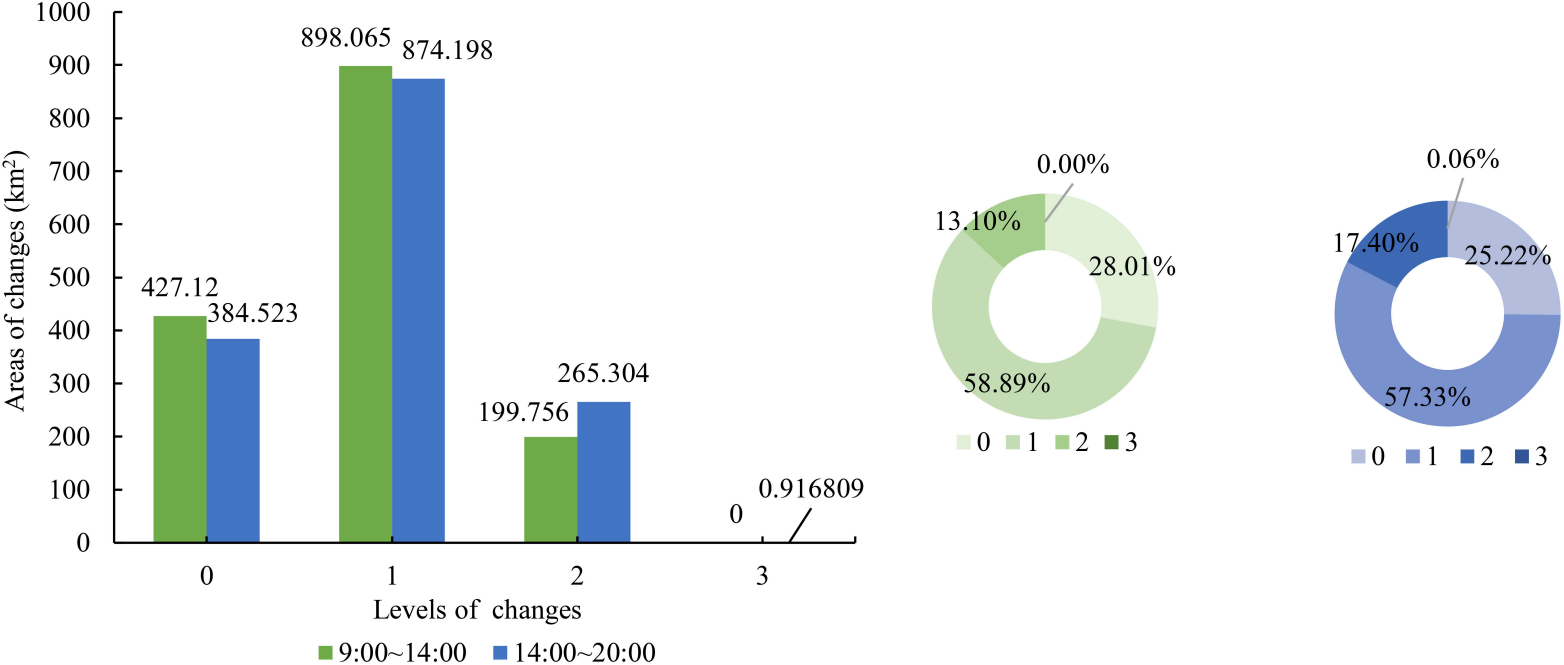
Areas of changes in UHV levels in Xiamen City on a hot day.

During the temperature rise from 09:00 to 14:00, the largest proportion of areas with exposure raised by 2 and 3 levels (80%) and UHV raised by 1 level (59%) were found in Xiamen City. During the temperature fall from 14:00 to 20:00, the largest proportion of areas with exposure decreased by 2 and 3 levels (78%) and UHV decreased by 1 level (57%) were found (Figures 7, 8).

**Figure 9:**
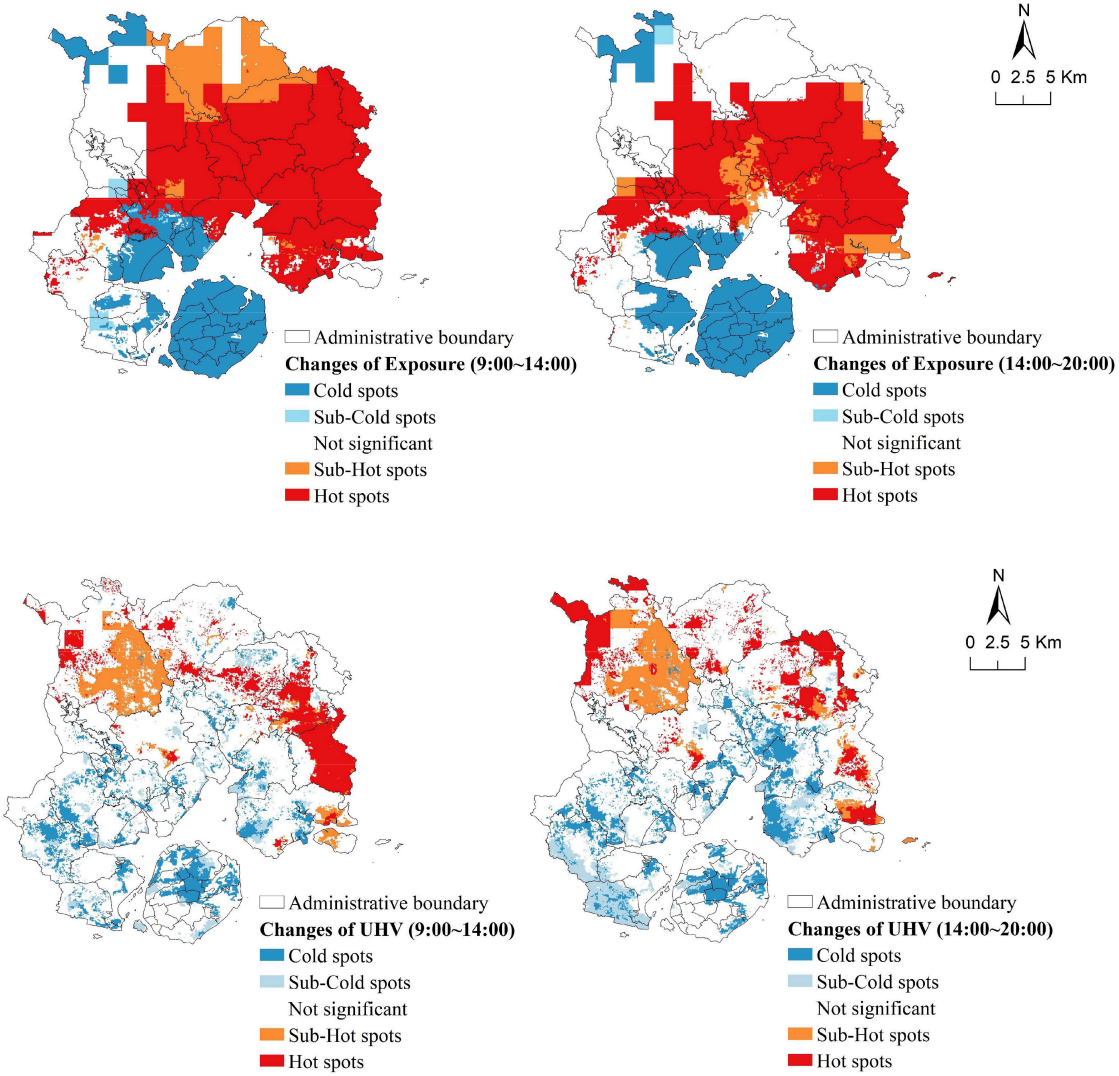
Hot spots of changes of exposure and UHV levels in Xiamen City on a hot day.

Further, a significant spatial clustering of changes of heat exposure and UHV was also observed (table is available in Supplementary Table 4). Figure 9 shows that in 09:00 to 14:00, Tong'an and Xiang'an were the hot spot areas of exposure increasing, and Lotus, Neicuo, and Xinxu were the hot spot areas of UHV increasing. The coastal streets of Jimei and Haicang, and Xiamen Island were the cold spot areas of exposure increasing, and the cold spot areas of UHV increasing were mainly distributed in the coastal, bay areas of Xiamen City, and the central part of Xiamen Island, especially the coastal streets of Songyu, Xinglin and Xingbin. In 14:00 to 20:00, the hot and cold spot areas of exposure and UHV decreasing were relatively consistent with the spatial distribution of those increasing in 09:00 to 14:00 of the same day.

### Spatial distribution characteristics of susceptibility in Xiamen City

The spatial structure of susceptibility in Xiamen City was “coastal-offshore-inland”, with an obvious spatial distribution characteristic of decreasing from coastal to inland. The above-mentioned spatial distribution characteristics were especially significant outside Xiamen Island, while the high susceptibility areas were mainly distributed in the northern and central parts of Xiamen Island, accounting for about 2/3 of the island's land area; the low-grade areas were mainly found in the southern part of Xiamen Island at higher elevations. It was found that the land cover type accounting for a relatively large proportion of the hot spots of susceptibility was impermeable surfaces, while that of the cold spots was mainly lakes and rivers (figure is available in Supplementary Figure 4).

### Spatial distribution characteristics of adaptive capacity in Xiamen City

The spatial distribution of adaptive capacity outside Xiamen Island was characterized by high in the southwest and low in the northeast, decreasing from the southwest to the northeast. Xiamen Island has a high level of adaptive capacity, its high population density, high level of disposable income per capita, and abundant public facilities, and consequently is also the hot spot areas for adaptive capacity (figure is available in Supplementary Figure 5).

### Geographical detection mechanism of urban heat vulnerability in Xiamen City

#### High and low vulnerability zoning

**Figure 10:**
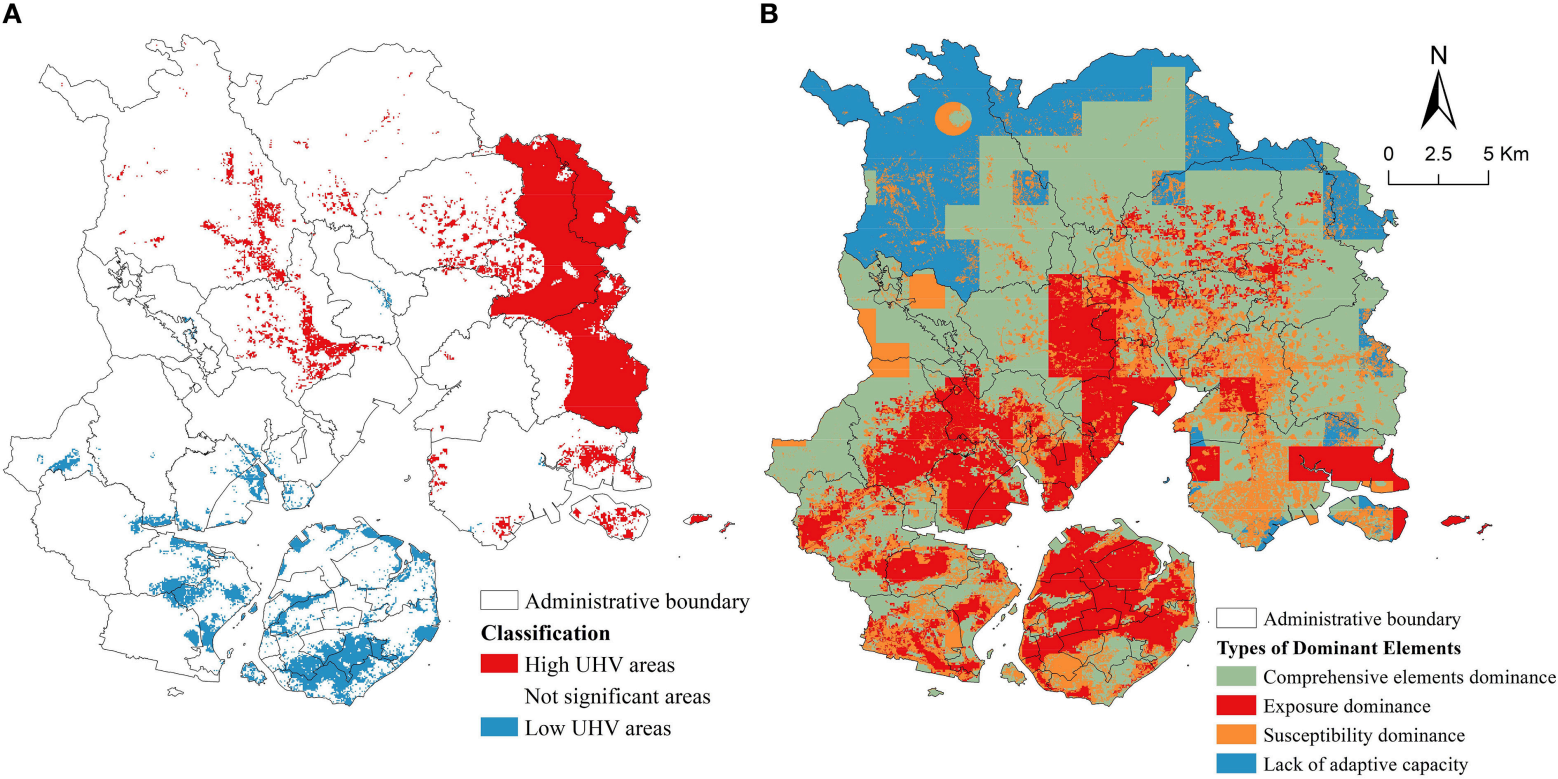
High and low UHV areas in Xiamen City **(A)**. Types of dominant elements of UHV in Xiamen City **(B)**.

In the summer of 2021, there were significant clustering areas of UHV in Xiamen City **(**table is available in Supplementary Table 5). Figure 10A shows that the southeastern part of Lotus in Tong'an, Xinxu, and Neicuo in Xiang'an were areas of significant clustering of high vulnerability (hot spots). The coastal areas of Maluan Bay and Xinglin Bay in Haicang, Yundang Lake and Wuyuan Bay Wetland Park on Xiamen Island, and the coastal areas around Xiamen Island were significant clusters of low vulnerability (cold spots).

#### Dominant element type zoning

Figure 10B shows that the dominant type of UHV in the coastal area of Xiamen City was dominated by “heat exposure”, the offshore area was dominated by “comprehensive elements”, and the northern part of Xiamen City was dominated by “lack of adaptive capacity”.

#### Geographical detection of the influencing factors

##### Geographic zoning

Table 3 shows the key influencing factors affecting the UHV of coastal, offshore, and inland areas of Xiamen City. Proportion of people with high school or higher education level had a strong explanatory power for the spatial differences in UHV in all areas of Xiamen; The cooling facility in the coastal zoning was the most important factor influencing UHV, and it was the proportion of population aged ≥ 65 years old in the offshore zoning, while in the inland zoning, disposable income per capita of the residents might change the regional UHV by influencing the residents' heat adaptive capacity.

**Table 3:** Results of factor detection for geographic zoning.

**Factors**	***q*** **Statistics**			
	**All areas of Xiamen City**	**Coastal zoning**	**Offshore zoning**	**Inland zoning**
TP*	**0.080981**	0.333947	**0.117215**	**0.240821**
HD*	**0.135117**	**0.140736**	**0.136611**	**0.272311**
THI*	**0.075942**	**0.276914**	**0.155905**	0.298722
POP*	**0.064354**	**0.161434**	**0.043175**	**0.040931**
LC*	**0.022149**	**0.053752**	**0.046135**	**0.105626**
DEM*	**0.068165**	**0.061133**	**0.01898**	**0.110234**
SL*	**0.036859**	**0.014707**	**0.025264**	0
WB*	**0.0265**	**0.020419**	**0.04144**	**0.015098**
POP_65*	0.397026	**0.173906**	0.461681	0.394743
POP_14*	0.229847	0.285346	0.349438	0.376784
HS*	0.478908	0.345327	0.350039	0.530435
IC*	0.330304	0.277712	0.280992	0.637035
CF*	0.204369	0.445104	0.226653	**0.013251**
HF*	**0.093209**	**0.17756**	**0.052565**	**0.007691**

Bolded values represent statistical significance at the 0.05 level; values marked in red represent the top 5 core influencing factors.

^*^Indicates that the indicator was unprocessed and input to the model using its original value. Refer to Table 1 for the definition of the factors.

##### Spatial statistical zoning

Table 4 shows the key influencing factors affecting the UHV of spatial statistical zoning of Xiamen City. Proportion of people with high school or higher education level had the strongest effect in both high and non-significant UHV areas, but in low HUV areas, the proportion of the population aged ≥ 65 years old was the strongest explanatory power for UHV.

**Table 4:** Results of factor detection for spatial statistical zoning.

**Factors**	***q*** **Statistics**		
	**High UHV** **areas**	**Low UHV** **areas**	**Non-significant** **areas**
TP*	0.223697	0.213432	**0.081265**
HD*	0.232726	0.204766	**0.117113**
THI*	0.163673	0.214357	**0.098408**
POP*	0.017893	0.013343	**0.054253**
LC*	**0.033942**	0.021034185	**0.034131363**
DEM*	**0.076437**	0.041854	**0.028741**
SL*	**0.012126**	0.005365	**0.026444**
WB*	0.001895	0.026246	**0.039255**
POP_65*	0.285403	0.268961	0.330962
POP_14*	**0.063215**	**0.163607**	0.290564
HS*	0.378804	0.215254	0.364148
IC*	**0.057463**	**0.177826**	0.336545
CF*	**0.015637**	**0.114271**	0.18811
HF*	0.001268	**0.191687**	**0.091644**

Bolded values represent statistical significance at the 0.05 level; values marked in red represent the top 5 core influencing factors.

^*^Indicates that the indicator was unprocessed and input to the model using its original value. Refer to Table 1 for the definition of the factors.

## Discussion

### The conceptual framework, index system, and assessment methods of urban heat vulnerability

Unlike previous studies that focus on the impact of socioeconomic and other human factors to construct models for evaluating social vulnerability (30, 40, 43, 48, 78), we took the vulnerability of the human-environmental system as the entry point, selected multiple natural and socioeconomic indicators characterizing the heat susceptibility of urban areas, emphasized the multi-scale adaptive capacity differences of urban systems, and constructed a conceptual framework for UHV. Our conceptual framework reflects the comprehensive impact of extreme heat on urban systems under multiple natural and human scale processes, and provides a theoretical perspective and analytical methodology for meteorological departments to further conduct more meteorological hazard impact assessments.

Previous heat vulnerability studies conducted at the local level using data from statistical yearbooks and historical weather stations have presented the results of multi-year vulnerability assessments with static effects (28, 46, 59, 61, 78). In terms of individual strategies to resist heat waves and government emergency actions, previous studies face a clear time-sensitive barrier, that is, they are limited by the lack of reference information for immediate decision making in response to extreme heat events, which makes it difficult for assessment results to support actions in community groups, households, and vulnerable populations (i.e., elderly, children) that lack heat adaptive capacity. This study used multiple sources of data, including dynamic high-resolution meteorological element products, the latest census data, and Map POI, combined with the UHV assessment method, effectively portrayed the comprehensive impact of heat hazards on urban areas, and finely characterized the spatial and temporal changes, distribution characteristics and patterns of UHV. Therefore, the UHV assessment method combining multi-source data not only addressed the “spatial and temporal dilemma” of policymakers and stakeholders in using assessment results in practice, but also contributed to gain insight into the cross-scale mechanisms driving the spatial change of UHV.

The heat vulnerability assessment combined with the population dynamic distribution model would be the direction to be further improved and deeply explored (79), and the barriers of accessing smaller-scale and more detailed indicators may be broken by the application of multi-source big data under the cooperation of multidisciplinary teams in the future. In summary, the assessment work conducted in Xiamen City shows that the conceptual framework and assessment methods proposed in this study can be quickly applied in other regions by combining geographical characteristics, and the assessment methods can be targeted and optimized by real local conditions.

### Spatial and temporal changes, distribution characteristics and patterns of urban heat vulnerability in coastal metropolis

Regardless of the hourly, daily, or monthly scales, we found that the exposure, susceptibility, adaptive capacity, and UHV of coastal metropolis had a spatial structure of “coastal-offshore-inland”. This “step-type” spatial structure of heat vulnerability is not the same as that of Fuzhou (28) and Chongqing (55), two hot cities in China, whose spatial structure of heat vulnerability is “circle-type”. The study we conducted in a coastal metropolis confirms and reinforces the importance of assessing the heat vulnerability of urban systems at the local level.

Related studies have assessed exposure, adaptive capacity, and vulnerability at the national or local level at multiple time points of 5 a or longer (48, 57), and currently we likely still lack knowledge of the spatial and temporal changes in UHV over shorter time periods (e.g., hot times of the day). We found that both exposure and UHV varied with the intensity of the exposure source (THI) throughout the day, but the change curve in UHV was more moderate compared to exposure. Moreover, it was also found that the magnitude of changes of heat exposure in Xiamen City was spatially greater than that of UHV. In recent vulnerability studies, exposure levels can be found to be modified by natural conditions and socioeconomic factors, for example, heat vulnerability in crowded areas of Punjab was found to be influenced by vegetation cover, housing density, and income (80); In Chongqing, a megalopolis in southwest China, different physical and social conditions contribute to differences in the spatial distribution of heat vulnerability in urban areas (55). Although the vulnerability of urban areas is influenced by exposure sources, regional vulnerability will be altered by the interaction of exposure, susceptibility, and adaptive capacity.

We also found hot and cold spot areas during UHV and exposure over time. The offshore areas of Xiamen City were significant hot spots of exposure changes, and the cold spots of UHV change were mainly distributed in the coastal and bay areas. In fact, the coastal zoning, especially Xiamen Island, has dense population, developed economic level and abundant infrastructure resources. This finding is similar to that observed in suburban and underdeveloped areas of Canada, where changes in socioeconomic vulnerability associated with heat risk are greater than in urban areas (58). Further, the change in exposure was not significant in northern part of Xiamen City, which may be related to the fact that this region is largely vegetation-covered mountainous areas, as the study conducted in Xiamen City has confirmed that green spaces experience less heat impact compared to urban areas (81). However, we also found that inland mountainous areas were significant hot spots for UHV, so all the above findings indicate that areas with well-developed socioeconomic conditions may be better equipped to maintain the relative stability of the urban system under heat exposure sources and multiple external disturbances. In other coastal metropolises like Xiamen, local governments and stakeholders should take advantage of their coastal geography location and well-developed economic and social resources to mitigate the heat vulnerability of urban systems by targeted measures (30, 52, 56).

### Geographical detection mechanism of urban heat vulnerability in coastal metropolis

Xiamen City has coastlines, numerous lakes, and dense river networks. Water bodies not only bring cooling effect to their surroundings (67, 68), but also affect the spatial distribution of their surrounding heat vulnerability (82), which was confirmed in our findings. We found that Maluan Bay, Wuyuan Bay, Yundang Lake and the coast of Xiamen Island were significant low UHV areas, in fact, during our local investigation, we found these areas are also livable areas with beautiful ecological environment in Xiamen City. In theoretical aspects, this finding affirms the importance of susceptibility of urban areas, especially water bodies in coastal metropolis, in shaping regional UHV. In practical aspects, we encourage urban planning departments in coastal metropolis to take advantage of their location near oceans, rivers, and water bodies to plan water-friendly squares or green walkways in densely populated areas, which may better mitigate the threat of heat waves and improve the adaptive capacity of the region.

Although the UHV in coastal areas of Xiamen City was mainly dominated by heat exposure, the results of factor detection found that the spatial distribution of UHV in coastal areas was less influenced by the composition factor of exposure compared with that of adaptive capacity. Similarly, inland areas, especially in the northern part of Xiamen, were vulnerable mainly due to lack of adaptive capacity, but these areas were non-significant areas of UHV. Further, we also found that the distribution of the elderly groups, education level, residents' per capita disposable income, and cooling facilities had strong explanatory power in influencing the spatial distribution of UHV. Research already points to aging as increasing heat vulnerability in the context of future climate warming (83), and income and cooling facilities have been identified as key factors in improving the heat adaptive capacity of Xiamen residents (71). These findings all indicate that the multi-scale adaptive capacity of urban areas can significantly modify the heat exposure of “coastal high, inland low” and reshape the spatial distribution of regional heat vulnerability. Moreover, we found that the clustering forces of high and low UHV areas mainly originated from the interaction of heat exposure sources and individual-level adaptive capacity, and the proportion of older groups and educated groups were stronger determinants of the forming process and the final pattern of UHV in Xiamen City. This finding reaffirms the important role of multi-scale adaptive capacity in shaping local UHV patterns. Investigations and research findings on household-level ability to withstand heat in India, South Asia (29) and Boston, North America (84) also indicated that the relationship between vulnerability and adaptive capacity deserves more focused attention. The above gives us the insight that people are the protagonists of cities, and people-centered decision making may need increased attention, prominent inclusion, and welfare provision for vulnerable groups in urban communities (85), which is important for building resilience of cities to disasters in the process of sustainable social development.

**Figure 11:**
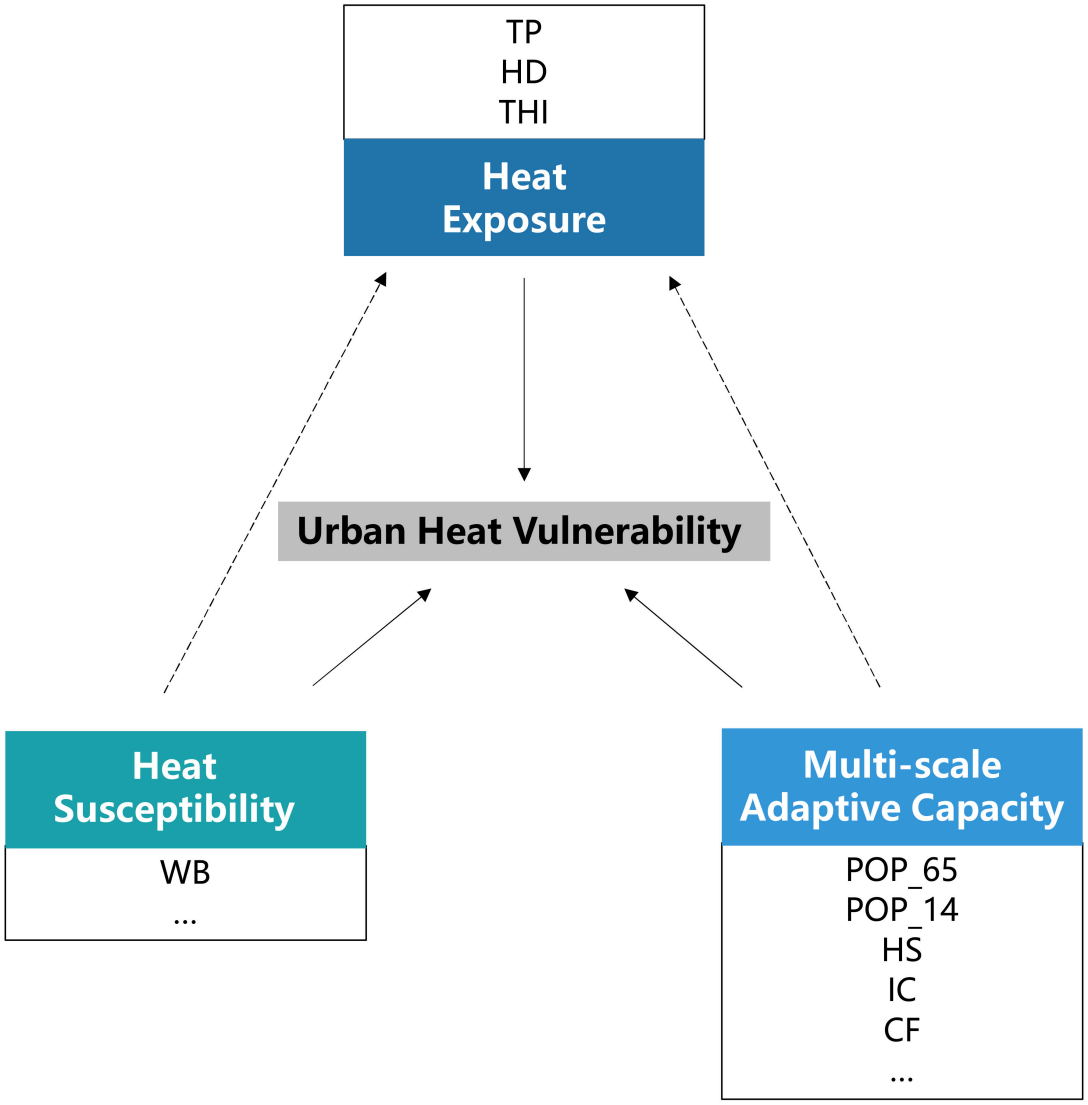
Map of the mechanism of UHV forming in Xiamen City. Refer to Table 1 for the definition of abbreviations. The solid arrows indicate that spatial and temporal changes in UHV were influenced by the interaction of exposure, susceptibility, and adaptive capacity. The dotted arrows indicate that heat susceptibility and multi-scale adaptive capacity consisting of natural environmental and socioeconomic conditions will interact with heat exposure to shape together the final UHV spatial distribution of urban areas. The box indicates that the indicators have a significant influence on the elements in Xiamen City; the ellipses in the box indicate that more influences may exist when the research framework is promoted to other study areas.

Finally, in this study of coastal metropolitan areas, susceptibility and multi-scale adaptive capacity consisting of natural environmental and socioeconomic conditions will interact with regional heat exposure to shape together the final UHV spatial distribution of urban areas (Figure 11). Nevertheless, the natural and human factors that influence UHV may not be consistent when the study is conducted in a specific region. The important purpose of proposing this conceptual framework is to call for more studies to explore the cross-scale mechanisms of local vulnerability forming from the human-environment systems perspective. This dynamic research framework deserves to be further developed.

## Limitations and prospects

Although it has been able to obtain statistical indicators to characterize adaptive capacity at the streets (towns) scale, this study still faces the limitation of obtaining inadequate information on adaptive capacity at the individual and household levels. Moreover, larger amounts of data involving physical and socioeconomic conditions are constrained by difficult accessibility and lack of processing power. We also did not compare the spatial and temporal change of UHV on monthly or annual scales, which may cause uncertainty in summarizing the general patterns of the findings.

In order to reduce the uncertainty of the model, we have rigorously selected the indicators to construct the index system of UHV. When meteorological bureaus and other departments apply this index system to conduct local assessments, they simply need to consult professionals in the relevant fields by issuing questionnaires to obtain the key parameters for modeling, and by operating GIS (geographic information system) software they can immediately assess and analyze local UHVs, or further develop UHV early warning systems that support stakeholders in decision making.

## Conclusion

In the 21st century, China's coastal regions have experienced large-scale rapid urbanization, and massive land development and utilization and cross-regional factor mobility have also increased instability and vulnerability of urban ecosystems. Urban areas, typical human-environment system, are complex in nature. With extreme heat events caused by global warming and intensified human activities on the land surface, the stability of various areas within the urban areas is uneven, which also makes local governments and residents exposed to potential economic losses and health threats. There is an urgent need to understand in depth the impact of extreme heat events under multiple external disturbances so that effective prevention and emergency management approaches can be adopted to reduce the loss of life and property.

This study constructed a conceptual framework and index system for UHV from the human-environment system perspective, and simulated the spatial and temporal characteristics of UHV under multi-scale natural and human processes in Xiamen City, a coastal metropolis, and revealed the cross-scale geographic detection mechanism for UHV forming. The results solve the “space-time dilemma” for decision makers and stakeholders in using the assessment results, and effectively support the government and planning departments in heat disaster emergency management. For Xiamen City, attention should be paid to the rational planning of water landscapes for the purpose of mitigating the threat of heat waves in crowded areas and improving the quality of the urban living environment.

## Data availability statement

The raw data supporting the conclusions of this article will be made available by the authors, without undue reservation.

## Author contributions

Conceptualization: WS. Methodology: WS and CW. Software, formal analysis, and writing—original draft preparation: CW. Investigation: WS, CW, YW, CX, and YH. Resources and data curation: ZH, CHW, and YHW. Writing—review and editing: CW, YW, CX, YH, and YZ. Visualization: CW and DZ. All authors have read and agreed to the published version of the manuscript.

## Funding

This research was funded by National Key Research and Development Plan Program of China (2016YFC0502905).

## Conflict of interest

Author DZ is a key member of this study and currently works for Fujian Zhitianqi Information Technology Co., Ltd. This study has no commercial or financial relationship with this company and received no funding from Fujian Zhitianqi Information Technology Co., Ltd. The remaining authors declare that the research was conducted in the absence of any commercial or financial relationships that could be construed as a potential conflict of interest.

## Publisher's note

All claims expressed in this article are solely those of the authors and do not necessarily represent those of their affiliated organizations, or those of the publisher, the editors and the reviewers. Any product that may be evaluated in this article, or claim that may be made by its manufacturer, is not guaranteed or endorsed by the publisher.

## Abbreviations

UHV: Urban Heat Vulnerability
THI: Temperature Humidity Index.
