# Supplementary material for: Urban heat vulnerability: A dynamic assessment using multi-source data in coastal metropolis of Southeast China
Source: Front Public Health. 2022 Oct 20;10:989963. doi: 10.3389/fpubh.2022.989963 (PMC9632749; doi:10.3389/fpubh.2022.989963)
Supplement: Supplementary file 1 [file Data_Sheet_1.PDF]

Supplementary Material

1 The Scientific Questions to be Addressed

Heat Vulnerability framework construction: Cutter (1) considered the natural environment and socioeconomic conditions at the local level and used factor analysis to construct the Hazards—of—Place of Vulnerability model, which is a classic result guided by the vulnerability framework under the perspective of the human-environmental system. Reid *et al*. (2) selected indicators including socioeconomic statistics, land use, and regional heat attendance or mortality rates, modeled them by principal component analysis, and mapped heat vulnerability at the community level, by which policymakers may take precise interventions for vulnerable groups and communities. However, the assessment methods of heat vulnerability are not unified due to the differences in regional geographical characteristics, the lack of data access and massive data processing power, and the fact that the integration of multi-source data has not yet been standardized. Above issues are also obstacles to the effective promotion of assessment methods at the local level.

Heat vulnerability assessment: Cutter and Finch (3) analyzed the changing spatial distribution of social vulnerability in the United States using census data from 1960 ~ 2000 and predicted social vulnerability for the future year 2010. This finding shows that the vulnerability of urban systems from a geographic perspective is not invariable. However, the statistical yearbook data based on administrative units has been unable to finely characterize the dynamic process of urban heat vulnerability under the influence of multiple external disturbances. In recent years, studies that integrate multi-source data including map Point of Interest (POI), remote sensing satellite imagery and related analytical techniques can effectively characterize the differences in the heat exposure pattern and multi-scale adaptive capacity (households, communities and regions) (4-6), which is meaningful for urban stakeholders to take differentiated measures to resist and reduce health and economic losses from heat hazards. However, these findings of heat vulnerability assessment still have problems like insufficient characterization accuracy and ex-post assessment cannot guide immediate emergency decisions. In addition, it remains lack of studies on the spatial and temporal change of heat vulnerability in specific cities, making it difficult to further generalize the common characteristics of urban heat vulnerability and its change patterns by comparing them with local cases.

Heat vulnerability mechanism: The classic studies by Johnson *et al*. (7) and Reid *et al*. (2) revealed the main environmental and socioeconomic factors influencing vulnerability through principal component analysis, but there is still a lack of insight into the mechanisms driving the spatial and temporal change of urban heat vulnerability under multiple external disturbances. Studies have identified significant temporal and spatial differences in well-being, economy, investment, infrastructure, ecosystem services, and social and cultural assets in the region, which may affect the spatial and temporal changes, distribution characteristics, and patterns of vulnerability of urban systems (7-12). Therefore, it is necessary to understand in-depth whether changes in natural and human elements amplify or diminish urban systems' heat vulnerability. For policy makers and stakeholders, understanding mechanisms of vulnerability formation can provide more effective reference for policy making and heat emergency management. Adaptive capacity, on the other hand, is a key element influencing urban heat vulnerability and has been widely mentioned (13). More

significantly, the assessment of the adaptive capacity of urban areas at multiple scales is important for sustainable city development and resilience building. Currently, the mechanisms of how key elements of the natural environment and socioeconomic conditions drive changes in heat vulnerability patterns remain unrevealed.

2 Characterization and Pre-processing of There Elements

Both the base data and the final product of this study were in raster format and all base data needs to be resampled to a resolution of 30 × 30 m after the normalization process has been completed.

2.1 Characterization of exposure and its component indicators

Exposure consists of exposure source and exposure quantity, where the exposure source requires an integration of both temperature and humidity indicators. This study used the Temperature Humidity Index (THI), which is used operationally by the China Meteorological Administration, to characterize the synergistic effect of temperature and humidity (Equation S1). THI has been widely used to reflect possible exposure risk from urban thermal environments by various national and local standards such as *the Evaluation of Climate Comfort of Habitat* (GB/T 27963- 2011) and *the Evaluation of Climate Comfort of Tourism* (DB46/T 461-2018) and related studies (14).

$$THI = T_i - 0.55(1 - Rh_i)(T_i - 14.4) \quad \text{(S1)}$$

Where T_i is the forecast maximum temperature (°C) at the i grid point; Rh_i is the forecast relative humidity (%) at the i grid point.

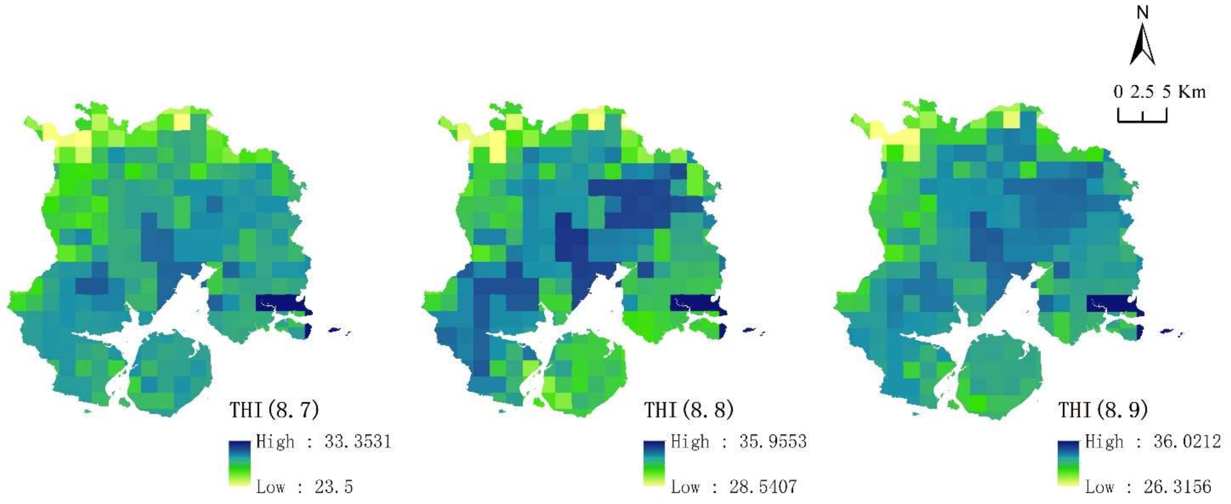

Figure A1| Spatial distribution of THI in Xiamen City from Aug. 7 to Aug. 9, 2021.

Population distribution uses the product of Stevens *et al.*'s (15) research achievements. Administrative boundaries are used as the basic unit of population statistics in China, so the spatial heterogeneity of geographical things within the statistical unit may be overlooked at small scales such as streets (towns), which may make it difficult to meet the demand for finely detailed spatial

characterization of heat exposure of the population. This study used a population spatial distribution simulation product that combines the advantages of remote sensing technology and big data technology, which is not only able to break through administrative boundaries, but also further increases the simulation accuracy of the data product to over 90% through natural and socioeconomic drivers such as night lighting, road network data, settlement data, and elevation. According to the assessment requirements, this study had obtained data products on the spatial distribution of population without administrative boundary adjustment through official channels (https://www.worldpop.org/) with a geographical resolution of 100 m × 100 m.

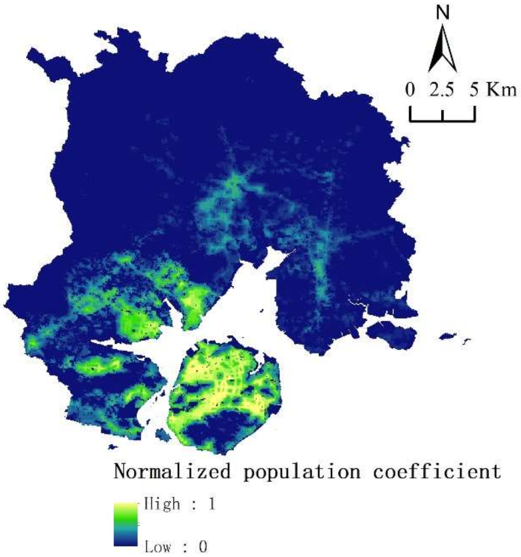

Figure A2| Spatial distribution of exposure coefficient of population distribution in Xiamen City.

2.2 Characterization of susceptibility and its component indicators

Multiple linear regression models are currently widely used to simulate and predict the spatial distribution of population (16). This study used population distribution to assign weights to different land cover types, reflecting the differences in the adverse effects of heat hazards on different human activities and economic and social couples in urban areas. Firstly, the resident population of the streets (towns) was used as a predicting variable, and the area of all land cover types within the boundary of each street (town) was calculated separately using ArcGIS 10.5 regional statistical tools and used as an explaining variable. This was followed by linear regression modelling using IBM SPSS 21.0 to select the main land cover types influencing population distribution; the main type factors were then used as explaining variables to build a multiple linear regression model with population distribution, and the best model with the largest linear trend fit value R^2 was used. Finally, the importance values of the selected land cover types in predicting population distribution were assigned as weights.

Population distribution tends to decrease with increasing elevation in China (17). In this study, the relationship between elevation and population density was investigated using the Digital Elevation Model (DEM). The weights of the effect of elevation on population distribution reflect the differences in the attributes of susceptibility of the human-environment system at different elevations. Firstly, the DEM of the study area was segmented at equal intervals according to the

actual situation using ArcGIS 10.5 Quantize method and reclassified tool; then the average population density in each elevation class was counted using the Spatial Analyst tool. Finally, linear, power, exponential, and quadratic functions were used to fit the average population density in each elevation interval, and the coefficients of the function model with a better fit were selected as the weights.

Water bodies or coastlines in the study area can be buffered using ArcGIS 10.5 buffer analysis tool (buffer distances need to be set according to local circumstances) to reflect differences in the magnitude of heat damage to the urban system within the buffer distance of different water bodies or coastline. It should be noted that when the assessment methodology of this study is applied to inland cities far from the coast, the coastline indicator needs to be removed or an indicator such as lakes, which are distinct from small water bodies and have a significant cooling effect, needs to be selected.

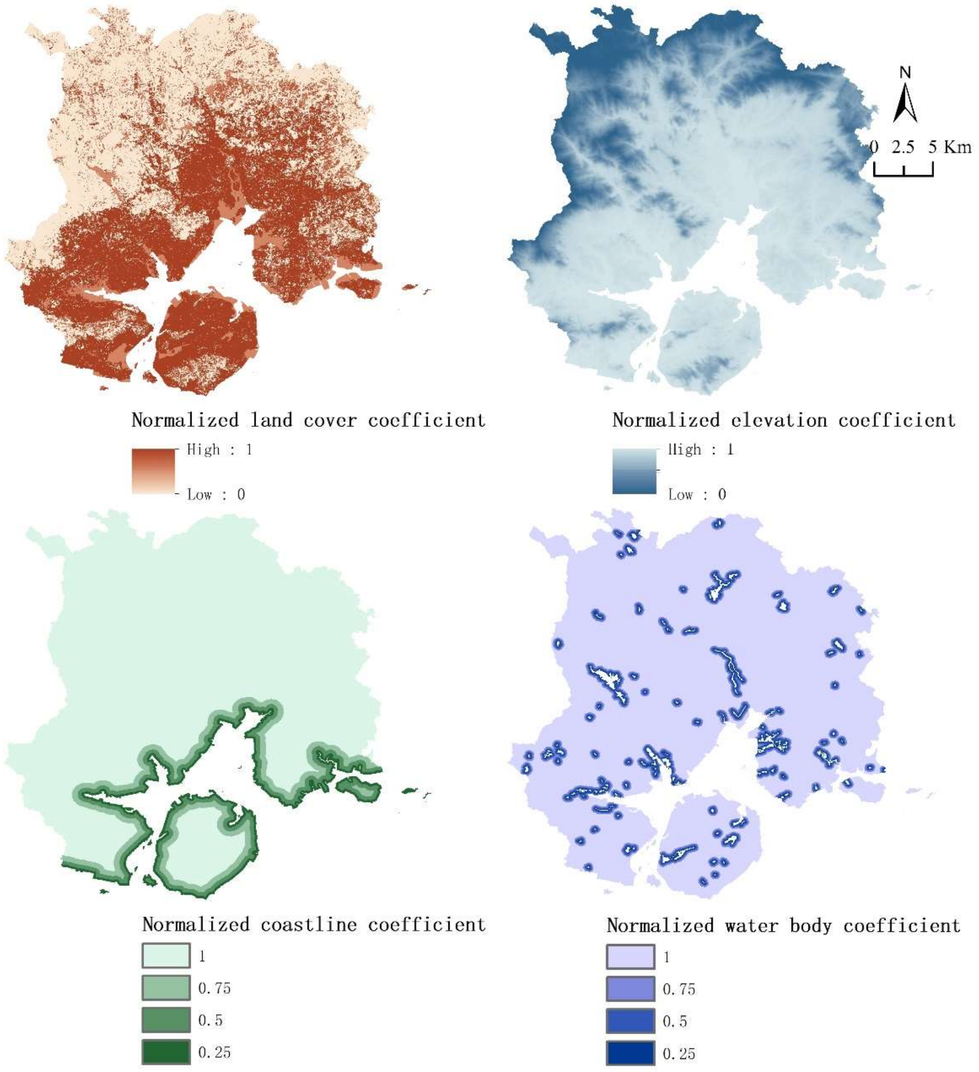

Figure A3| Spatial distribution of susceptibility coefficients of landcover, elevation, sealine, and water body in Xiamen City.

2.3 Characterization of adaptive capacity and its component indicators

With reference to the findings of the large-sample questionnaire survey work already conducted by our research team in Xiamen City (13) and Fuzhou City (18), combined with the requirement for refined spatial characterization, firstly, individual adaptive capacity data were used from the 7th National Census data counted at the streets (towns) level. Due to data accessibility constraints, per capita disposable income of residents was adopted from the latest census data collected at the administrative district level. The above basic data were converted to raster layers by ArcGIS 10.5. Secondly, POIs for the cooling and medical facilities were obtained by programming Python scripts. Because different levels and types of infrastructure have different service areas, this study used the ArcGIS 10.5 buffer zone tool to make a service area layer for each type of facility site in the study area, and to calculate the number of overlaps between the service areas of cooling and medical facilities. The number of overlaps reflects the differences in the ability of urban areas to adapt to extreme heat stress.

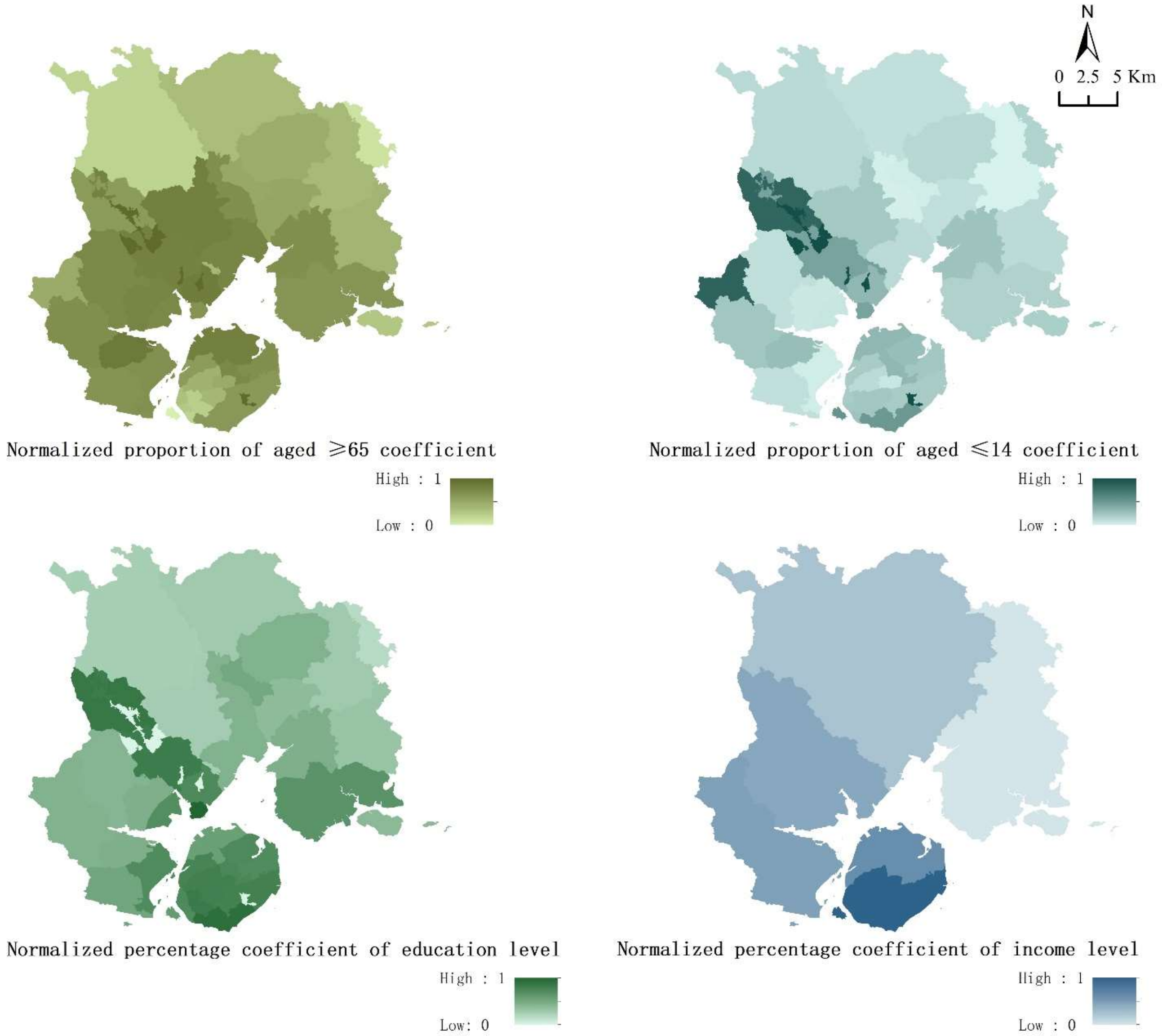

Figure A4| Spatial distribution of adaptive capacity coefficients of individual-level indicators in Xiamen City.

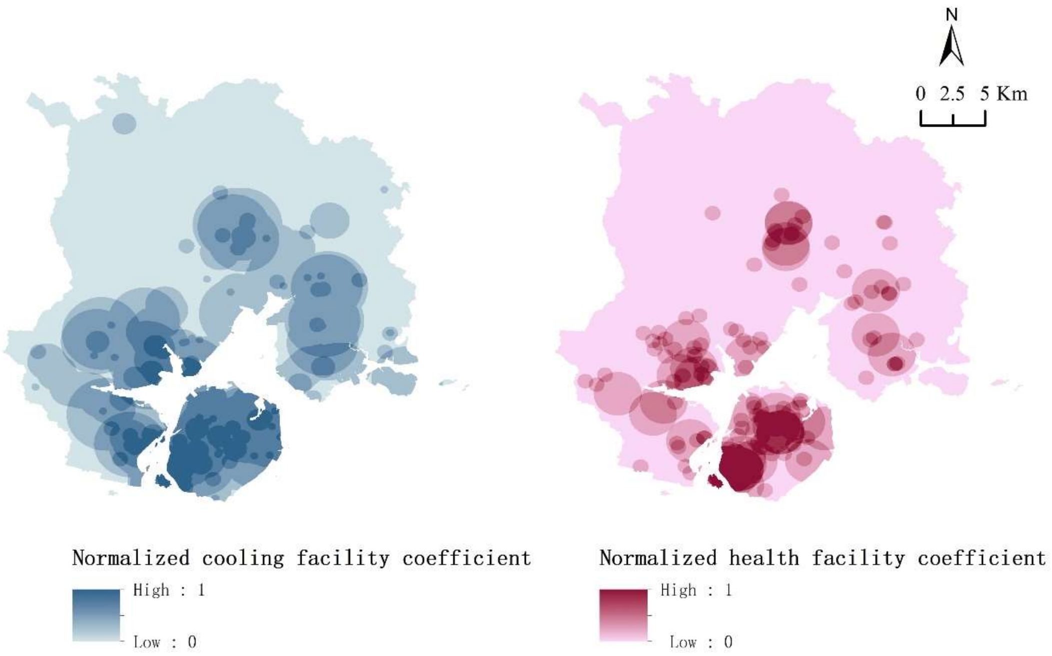

Figure A5| Spatial distribution of adaptive capacity coefficients of regional-level indicators in Xiamen City.

3 Weights of the Component Indicators of Three Elements

In this study, a group decision questionnaire was distributed to 10 experts from relevant fields, climate research institutes, planning and consulting departments and government research offices (We carried out the distribution of the expert group decision questionnaire in June 2021, which is 5 experts from Fujian Meteorological Bureau, 3 researchers from the field of heat hazard research in Xiamen University, Fujian Normal University, Nanjing University, and 2 from government policy-making departments in Fuzhou City and Xiamen City). Calculations of expert group decisions showed that the weights of the internal indicators of exposure were 0.8100 for THI and 0.1900 for POP; the weights of the internal indicators of susceptibility were 0.5054 for LC, 0.1169 for DEM, 0.1561 for CL, and 0.2216 for distance from WB; the weights of the internal indicators of adaptive capacity were 0.1012 for POP_65, 0.0701 for POP_14, 0.0771 for HS, 0.2597 for IC, 0.2981 for CF, 0.1939 for HF. After adjusting the judgment matrix that does not meet the consistency and supplementing the residual matrix, the decision results of all experts finally met the consistency < 0.1, which means that the weights determined by the judgment matrix are suitable for the internal indicator integration of the elements of exposure, susceptibility, and adaptive capacity.

4 The discrete results of the influencing factors

Common discretization methods are equal, natural, quantile, geometric, standard deviation, and manual. The interpretation of each classification method can be found in the ArcGIS Pro instructions (19). Table A1 lists the optimal classification solutions for the potential influencing factors that require discrete processing. The rest of the potential influencing factors are processed as follows: LC kept at its original value, CL and WB reclassified into 4 classes, IC reclassified into 6 classes

according to the amount of the value, CF and HF reclassified into 5 classes and 16 classes according to the original overlap number of buffer zones.

Table A1. The optimal classification solution for potential influencing factors that require to be discrete

**Potential influencing factors**	**TP**	**HD**	**THI**	**POP**
**Optimal classification solution**	natural	natural	natural	quantile
**Number of classifications**	20	19	18	20
**Potential influencing factors**	**DEM**	**POP_65**	**POP_14**	**HS**
**Optimal classification solution**	natural	natural	natural	natural
**Number of classifications**	13	19	12	17

5 Supplementary Figures and Tables

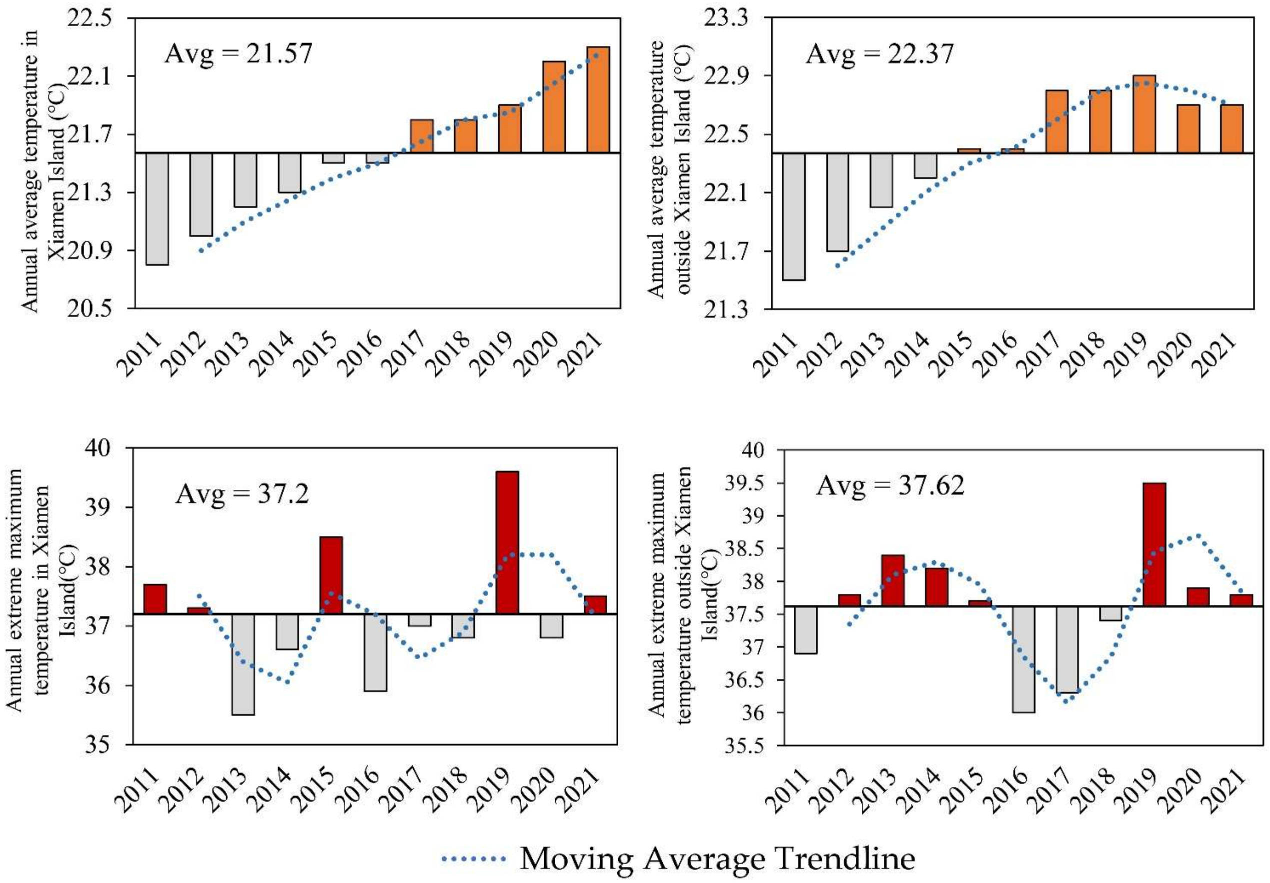

Supplementary Figure 1| Trends of annual average temperature and annual extreme maximum temperature in Xiamen City from 2011 to 2021. Source: Xiamen Annual Climate Report (2011~2021).

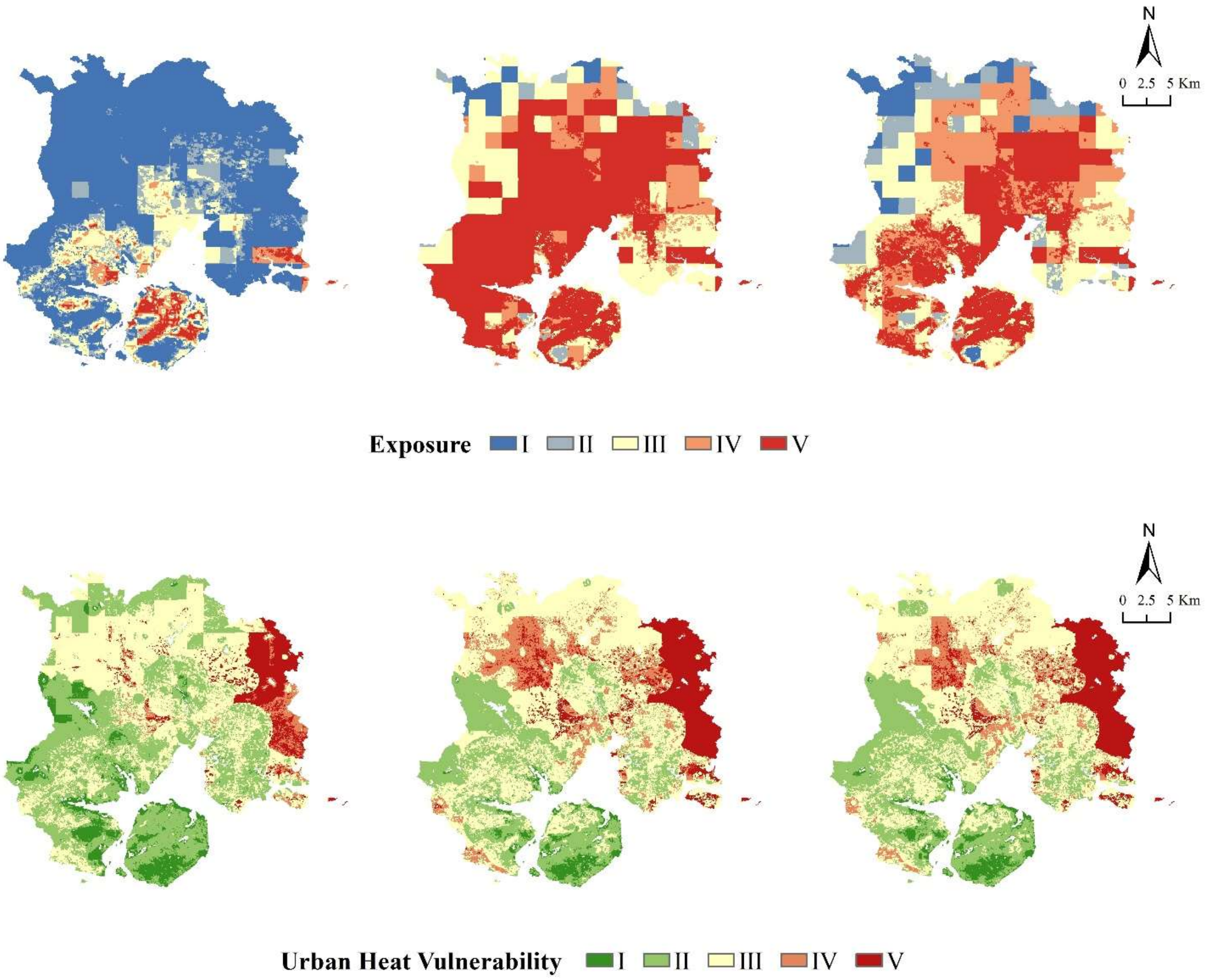

Supplementary Figure 2| Spatial distribution of exposure and UHV in Xiamen City from Aug. 7 ~ Aug. 9, 2021.

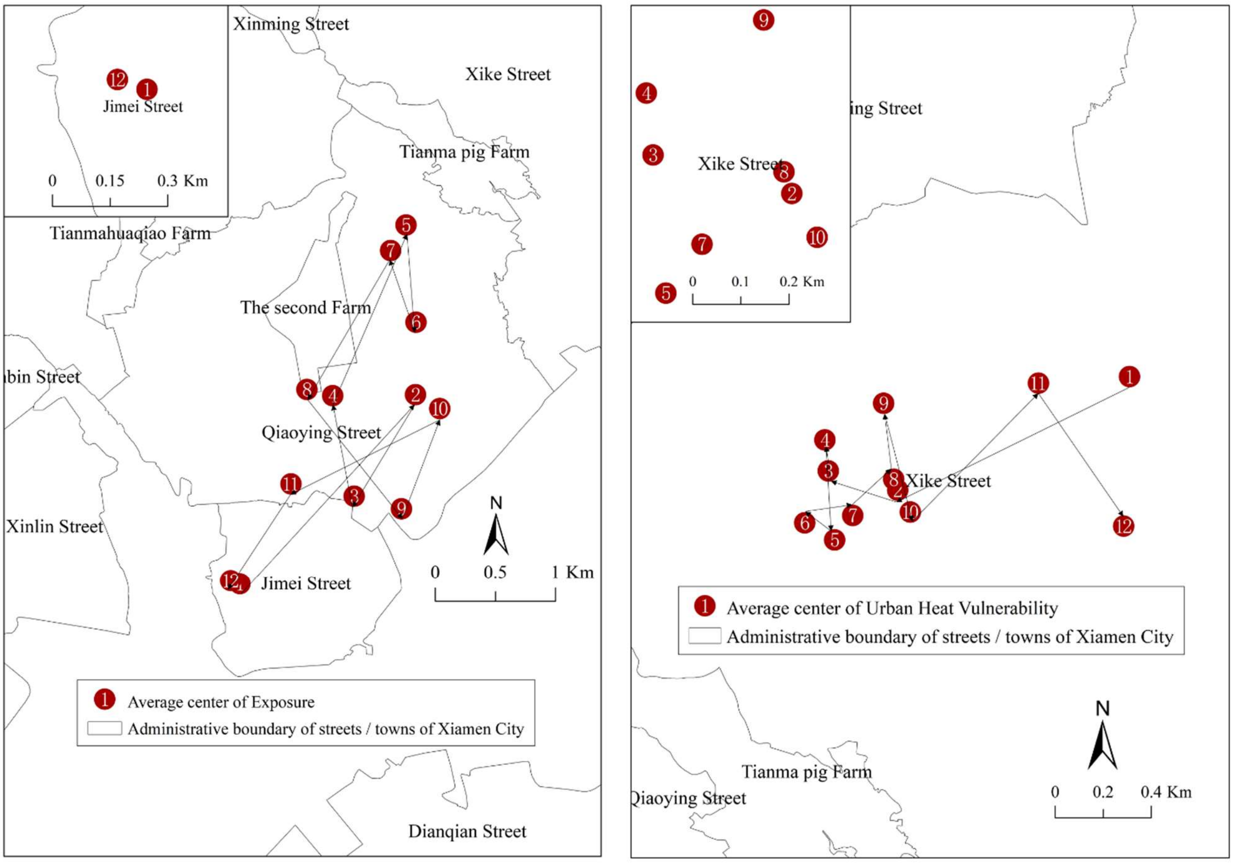

Supplementary Figure 3| Spatial dynamics of the average center of exposure and UHV in Xiamen City on a hot day.

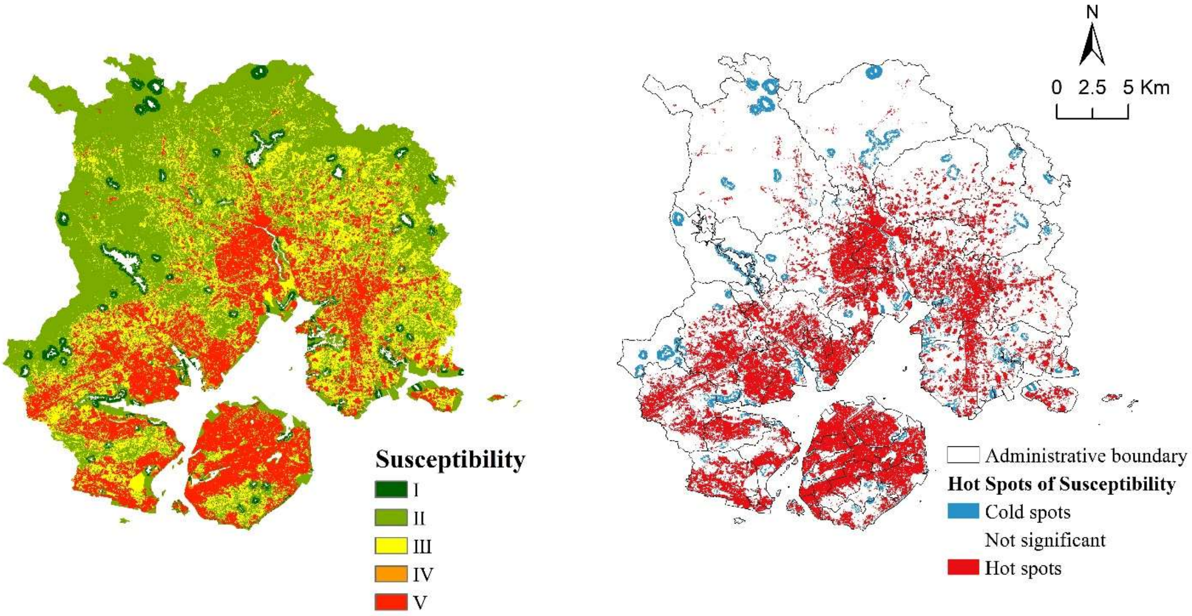

Supplementary Figure 4| Spatial distribution of susceptibility levels and hot spots in Xiamen City.

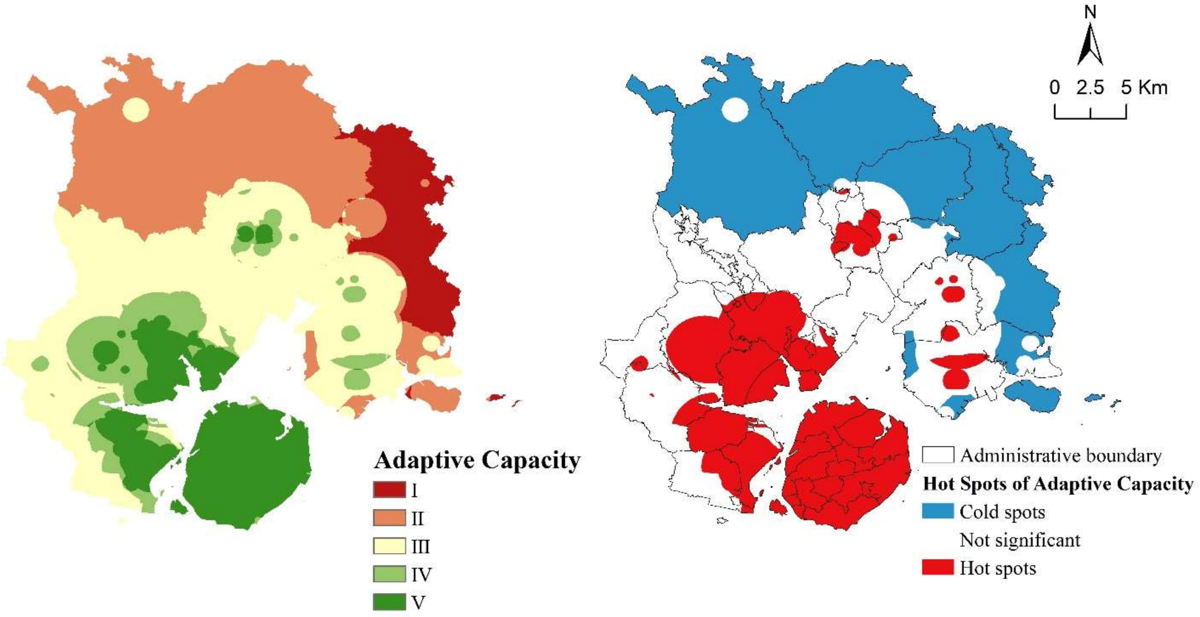

Supplementary Figure 5| Spatial distribution of susceptibility levels and hot spots in Xiamen City.

Supplementary Table 1 | Data sources and descriptions.

Data	Description	Types	Spatial Resolution	Sources
Dynamic weather element numerical forecast data	Numerical forecasting products of grid meteorological elements of temperature and humidity produced by FZ-MOS system	GeoTiff	2.5 km	FZ-MOS System of Fujian Provincial Meteorological Bureau
Landcover	2020 Global 30-m land-cover dynamic monitoring products with fine classification system	GeoTiff	30m	The official website of Earth Big Data Science Project (https://data.casearth.cn/)
Elevation	Global 12.5m Digital Elevation Model (DEM) products	GeoTiff	12.5m	Alaska Satellite Facility (https://asf.alaska.edu/)
Coastline	Coastline vector data of Xiamen City	Shapefile	-	Xiamen Natural Resources Bureau
Waterbody	Water body vector data of Xiamen City	Shapefile	-	Xiamen Natural Resources Bureau
Administrative Boundary	Administrative boundary data of streets (towns) of Xiamen City	Shapefile	-	Xiamen Natural Resources Bureau

Population Distribution	Global 100m resolution population distribution data product	GeoTiff	100m	Worldpop (https://www.worldpop.org/)
The 7th national census data	Number of resident populations in streets (towns) in Xiamen City	Shapefile	-	Xiamen Statistics Bureau
	Number of resident populations by age group in streets (towns) in Xiamen City	Shapefile	-	Xiamen Statistics Bureau
	Number of resident populations by education level group in streets (towns) in Xiamen City	Shapefile	-	Xiamen Statistics Bureau
	Per capita disposable income of residents at the administrative district level of Xiamen City	Shapefile	-	Xiamen Statistics Bureau
Point of Interest Data	POI with cooling function in Xiamen City	Shapefile	-	AutoNavi (https://ditu.amap.com/)
	POI with medical & health function in Xiamen City	Shapefile	-	AutoNavi (https://ditu.amap.com/)
	POI of various scenic spots in Xiamen City	Shapefile	-	AutoNavi (https://ditu.amap.com/)

Supplementary Table 2| Average and standard deviation of exposure levels for Xiamen City and its six districts during a heat wave.

Date		Xiamen	Xiang'an	Tong'an	Siming	Jimei	Huli	Haicang
2021.08.07	Avg	1.64	1.48	1.32	2.54	1.97	3.41	1.65
	Std.	1.05	0.97	0.67	1.49	1.12	1.38	0.93
2021.08.08	Avg	4.16	3.86	4.03	4.16	4.57	4.67	4.49
	Std.	1.09	0.96	1.26	1.02	0.79	0.70	0.86
2021.08.09	Avg	3.64	3.69	3.44	3.86	3.73	4.60	3.72
	Std.	1.25	1.01	1.40	1.20	1.20	0.77	1.11

Supplementary Table 3| Average and standard deviation of vulnerability levels for Xiamen City and its six districts during a heat wave.

Date		Xiamen	Xiang'an	Tong'an	Siming	Jimei	Huli	Haicang
2021.08.07	Avg	2.62	3.46	2.73	1.53	2.06	1.80	2.04
	Std.	0.97	1.17	0.63	0.50	0.59	0.43	0.69
2021.08.08	Avg	3.07	3.86	3.31	1.68	2.36	2.22	2.50
	Std.	1.01	1.13	0.68	0.55	0.53	0.66	0.72
2021.08.09	Avg	2.98	3.82	3.19	1.63	2.29	2.16	2.36
	Std.	1.01	1.15	0.66	0.54	0.51	0.64	0.71

Supplementary Table 4| Results of spatial autocorrelation of the changes of exposure and UHV levels.

		Moran's I	Z-score	P value	Clustering Patterns
**Exposure**	**09:00~14:00**	0.446112	115.150008	$p < 0.001$	aggregation
	**14:00~20:00**	0.456981	29.521337	$p < 0.001$	aggregation
**UHV**	**09:00~14:00**	0.2795	100.0475	$p < 0.001$	aggregation
	**14:00~20:00**	0.3123	108.8680	$p < 0.001$	aggregation

Supplementary Table 5| Results of spatial autocorrelation of UHV levels in Xiamen City.

Moran's I	Z-score	P-value	Clustering Patterns
0.5473	171.0487	$p < 0.001$	aggregation

6 Reference

1. Cutter SL. Social Vulnerability to environmental hazards. *Prog Hum Geog*. (1996) 20: 529-39. doi: 10.1177/030913259602000407
2. Reid CE, O'Neill MS, Gronlund CJ, Brines SJ, Brown DG, Diez-Roux AV, et al. Mapping Community Determinants of Heat Vulnerability. *Environ Health Persp*. (2009) 117: 1730-6. doi: 10.1289/ehp.0900683
3. Cutter SL, Finch C. Temporal and spatial changes in social vulnerability to natural hazards. *Proceedings of the National Academy of Sciences*. (2008) 105: 2301-6. doi: 10.1073/pnas.0710375105
4. Weber S, Sadoff N, Zell E, de Sherbinin A. Policy-relevant indicators for mapping the vulnerability of urban populations to extreme heat events: A case study of Philadelphia. *Appl Geogr*. (2015) 63: 231-43. doi: 10.1016/j.apgeog.2015.07.006
5. Nandy S, Singh C, Das KK, Kingma NC, Kushwaha SPS. Environmental vulnerability assessment of eco-development zone of Great Himalayan National Park, Himachal Pradesh, India. *Ecol Indic*. (2015) 57: 182-95. doi: 10.1016/j.ecolind.2015.04.024
6. Uejio CK, Wilhelmi OV, Golden JS, Mills DM, Gulino SP, Samenow JP. Intra-urban societal vulnerability to extreme heat: The role of heat exposure and the built environment, socioeconomics, and neighborhood stability. *Health Place*. (2011) 17: 498-507. doi: 10.1016/j.healthplace.2010.12.005
7. Johnson DP, Stanforth A, Lulla V, Luber G. Developing an applied extreme heat vulnerability index utilizing socioeconomic and environmental data. *Appl Geogr*. (2012) 35: 23-31. doi: 10.1016/j.apgeog.2012.04.006
8. Abrar R, Sarkar SK, Nishtha KT, Talukdar S, Shahfahad, Rahman A, et al. Assessing the Spatial Mapping of Heat Vulnerability under Urban Heat Island (UHI) Effect in the Dhaka Metropolitan Area. *Sustainability-Basel*. (2022) 14. doi: 10.3390/su14094945
9. Bradford K, Abrahams L, Hegglin M, Klima K. A Heat Vulnerability Index and Adaptation Solutions for Pittsburgh, Pennsylvania. *Environ Sci Technol*. (2015) 49: 11303-11. doi:

10.1021/acs.est.5b03127
10. El-Zein A, Tonmoy FN. Assessment of vulnerability to climate change using a multi-criteria outranking approach with application to heat stress in Sydney. *Ecol Indic*. (2015) 48: 207-17. doi: 10.1016/j.ecolind.2014.08.012
11. Ho HC, Knudby A, Chi G, Aminipouri M, Lai DY. Spatiotemporal analysis of regional socio-economic vulnerability change associated with heat risks in Canada. *Appl Geogr*. (2018) 95: 61-70. doi: 10.1016/j.apgeog.2018.04.015
12. Huang X, Li Y, Guo Y, Zheng D, Qi M. Assessing Urban Risk to Extreme Heat in China. *Sustainability-Basel*. (2020) 12. doi: 10.3390/su12072750
13. Wu C, Shui W, Yang H, Ma M, Zhu S, Liu Y, et al. Heat Adaptive Capacity: What Causes the Differences Between Residents of Xiamen Island and Other Areas? *Frontiers in Public Health*. (2022) 10: 799365. doi: 10.3389/fpubh.2022.799365
14. Hasanah NAI, Maryetnowati D, Edelweis FN, Indriyani F, Nugrahayu Q. The climate comfort assessment for tourism purposes in Borobudur Temple Indonesia. *Heliyon*. (2020) 6: e5828. doi: 10.1016/j.heliyon.2020.e05828
15. Stevens FR, Gaughan AE, Linard C, Tatem AJ. Disaggregating Census Data for Population Mapping Using Random Forests with Remotely-Sensed and Ancillary Data. *Plos One*. (2015) 10: e107042. doi: 10.1371/journal.pone.0107042
16. Arraras JI, Nolte S, Liegl G, Rose M, Manterola A, Illarramendi JJ, et al. General Spanish population normative data analysis for the EORTC QLQ-C30 by sex, age, and health condition. *Health Qual Life Out*. (2021) 19: 208. doi: 10.1186/s12955-021-01820-x
17. Chen M, Xu CG, Wang RS. Key natural impacting factors of China's human population distribution. *Popul Environ*. (2007) 28: 187-200. doi: 10.1007/s11111-007-0041-x
18. Shui W, Chen Z, Deng J, Li Y, Wang Q, Wang W, et al. Evaluation of urban high temperature vulnerability of coupling adaptability in Fuzhou, China (In Chinese). *Acta Geographica Sinica*. (2017) 72: 830-49. doi: 10.11821/dlxb201705006
19. ESRI. *Data Classification Methods*. Available online at: https://pro.arcgis.com/zh-cn/pro-app/latest/help/mapping/layer-properties/data-classification-methods.htm (accessed March 4, 2022).
